# Claudin-5a is essential for the functional formation of both zebrafish blood-brain barrier and blood-cerebrospinal fluid barrier

**DOI:** 10.1186/s12987-022-00337-9

**Published:** 2022-06-03

**Authors:** Yanyu Li, Chunchun Wang, Liang Zhang, Bing Chen, Yuqian Mo, Jingjing Zhang

**Affiliations:** 1 Affiliated Hospital of Guangdong Medical University & Key Laboratory of Zebrafish Model for Development and Disease of Guangdong Medical University, Zhanjiang, 524001 China; 2 College of Life Sciences, Shandong Normal University, Jinan, 250014 China; 3 School of Public Health, Guangdong Medical University, Dongguan, 523808 China; 4 The Marine Biomedical Research Institute of Guangdong Zhanjiang, Zhanjiang, 524023 China

**Keywords:** Claudin-5, Blood-brain barrier, Blood-cerebrospinal fluid barrier, Zebrafish, Tight junction

## Abstract

**Background:**

Mammalian Claudin-5 is the main endothelial tight junction component maintaining blood-brain barrier (BBB) permeability, while Claudin-1 and -3 seal the paracellular space of choroid plexus (CP) epithelial cells contributing to the blood-cerebrospinal fluid barrier (BCSFB). In zebrafish, two paralogs of *claudin-5a* and *-5b* are expressed while their roles in the formation of BBB and BCSFB are unclear.

**Methods:**

The expression patterns of Claudin-5a and -5b in zebrafish brains were systematically analyzed by immunofluorescence (IF) assay. The developmental functions of Claudin-5a and -5b were characterized by generating of *claudin-5a* and *-5b* mutants respectively. Meanwhile, the cerebral inflammation and cell apoptosis in *claudin-5a*^*-/-*^ were assessed by live imaging of transgenic zebrafish, RT-qPCR, IF, and TUNEL assay. The integrity of BBB and BCSFB was evaluated by in vivo angiographic and dye permeation assay. Finally, RT-qPCR, whole-mount RNA in situ hybridization (WISH), and transmission electron microscopy (TEM) analyses were performed to investigate the development of cerebral vessels and choroid plexus.

**Results:**

We showed that Claudin-5a and -5b are both expressed in zebrafish cerebrovascular endothelial cells (ECs). In addition, Claudin-5a was strongly expressed in CP epithelial cells. Loss of Claudin-5b showed no effect on zebrafish vasculogenesis or BBB function. In contrast, the knockout of *claudin-5a* caused a lethal phenotype of severe whole-brain oedema, ventricular dilatation, and cerebral hernia in zebrafish larvae, although the cerebral vasculogenesis and the development of CP were not altered. In *claudin-5a*^*-/-*^ , although ultrastructural analysis of CP and cerebral capillary showed intact integrity of epithelial and endothelial tight junctions, permeability assay indicated a disruption of both BBB and BCSFB functions. On the molecular level, it was found that ZO-1 was upregulated in the CP epithelium of *claudin-5a*^*-/-*^, while the notch and shh pathway responsible for CP development was not affected due to loss of Claudin-5a.

**Conclusions:**

Our findings verified a non-functional role of zebrafish Claudin-5b in the BBB and identified Claudin-5a as the ortholog of mammalian Claudin-5, contributing to the development and the functional maintenance of both BBB and BCSFB.

**Supplementary Information:**

The online version contains supplementary material available at 10.1186/s12987-022-00337-9.

## Background

The barriers system of the central nervous system (CNS) mainly include the blood-brain barrier (BBB), blood-cerebrospinal fluid barrier (BCSFB), and arachnoid barrier, which are responsible for the maintenance of the neuronal microenvironment by isolating the brain from systemic blood circulation or cerebrospinal fluid (CSF) [1]. Due to its avascular property, the arachnoid epithelial barrier does not serve as an interface for exchange between the CNS [1]. BBB separates circulating blood from the brain parenchyma and is localized at cerebral capillary endothelial cells (ECs) which form intercellular tight junctions (TJs) to exclude detrimental blood molecules [2]. BCSFB, an epithelial barrier, is consisted of choroid plexus (CP) epithelial cells which are tightly connected by apical TJs to prevent paracellular permeability of water-soluble molecules and preserve the homeostasis of CSF [3]. Brain barriers dysfunction is implicated in many CNS diseases, such as stroke, infectious disease, traumatic brain injury (TBI), neurodegenerative disease, multiple sclerosis, primary or secondary tumors, schizophrenia, and epilepsy [3, 4]. Elucidating the molecular mechanisms regulating the development of brain barriers will provide strategies for the therapy of CNS diseases.

Zebrafish is a good model for studying the development of vertebrates and for large-scale screening of new drugs [5, 6]. The structure and function of zebrafish brain barriers are conserved to higher vertebrates [7–10]. The constituents of neurovascular units and CNS barriergenesis of zebrafish are similar to most mammalians suggesting that zebrafish is a powerful and ideal system to study the development and pathology of the brain barriers. Nevertheless, the detailed mechanisms regulating the formation and maintenance of zebrafish brain barriers are not yet well characterized [11].

The TJs of brain microvascular ECs or cuboidal CP epithelial cells are located at the most apical part of junctional complexes to form a paracellular barrier that restricts the leakage of most water-solute molecules and ions. TJs are composed of transmembrane molecules (Claudins, Occludins, and JAMs), cytoplasmic adaptors (ZOs, Cingulin, Jacop, MAGIs, and MPPs), and cytoskeletal proteins [3, 12]. Claudins (CLDNs) are one of the most important TJ proteins consisted of four transmembrane regions, two extracellular loops (ECLs), a short intracellular turn, cytoplasmic N- and C-terminal regions, and there exist more than 27 CLDN family members in mammals [13, 14]. Different CLDNs are expressed in distinct tissues and play diverse physiological functions, such as sealing gaps (CLDN-1, -3, -4, -5, -7, -11) and forming channels (CLDN-2, -10a, -10b, -15) [13, 14].

CLDN5 is highly expressed in the ECs of mammalian CNS capillaries [15, 16], and plays a critical role in restricting the paracellular diffusion of small molecules [17]. Exogenous expression of CLDN5 in cultured rat brain capillary ECs strengthens barrier function [18]. Nitta et al. generated *CLDN5* mutant mice and reported that the absence of CLDN5 could not affect the development and morphology of cerebral vessels while causing a size-selective leakage of the BBB to molecules with a weight up to 800 Da [19]. After exposure to skin under low dose UVB, the lymphatic vessel in *CLDN5*^+/-^ mice was leaky, and oedema and inflammation were exacerbated compared with that in wild type mice [20].

In zebrafish, two paralogs of *cldn5a* and *cldn5b* are expressed with distinct mRNA expression patterns [21]. *cldn5a* is mainly expressed in the CNS and arteries while *cldn5b* is only distributed in the arterial vascular system [22, 23]. van Leeuwen et al. has generated *cldn5a* transgenic zebrafish with expression in cerebral vessels and CP [9]. However, the protein expression pattern of zebrafish Cldn5s during late development is not elucidated. By analyzing the genomic region and closer gene relatives of *cldn5a* and *cldn5b*, it indicates that zebrafish *cldn5a* shared synteny with human *Cldn5* [9]. Zhang et al. has found that Cldn5a is essential for embryonic brain ventricle expansion by forming a neuroepithelial barrier [23], and Ahn et al. has reported that *cldn5a* is required for maintaining the integrity of the zebrafish blood-neural barrier [24]. However, the role of *cldn5b* in the formation of the embryonic barrier is still unclear. Despite the size-selective leakage of BBB identified in *CLDN5*-deficient mice, the development and morphology of blood vessels were not altered [19]. In our previous study, we found that *cldn5a* limits EC motility and is required for the lumenization of the dorsal aorta [25]. Nevertheless, whether *cldn5a* affects the normal development of cerebral vasculature and CP epithelia is unclear.

Here, we firstly explored the expression of CLDNs or Cldn5s in cerebral vessels and CP epithelium of human, mice, and zebrafish brains respectively, and systematically analyzed the development roles of zebrafish Cldn5a and Cldn5b by generating *cldn5a* and *cldn5b* mutants. We found that *cldn5a* and *cldn5b* showed distinct expression patterns in zebrafish CNS, while neither of them had functional complementation on each other on the functional formation of CNS barriers. Although both Cldn5s express in cerebral vessels, only Cldn5a was detected being expressed in CP epithelium. Moreover, we further determined that *cldn5a* but not *cldn5b* was essential for the formation and functional maintenance of the BBB and BCSFB by analyzing the paracellular permeability using different size tracers. Finally, except for the upregulation of ZO-1 in CP epithelial cells, we verified that the formation of CP and cerebral vessels was not affected due to the loss of *cldn5a*, by analyzing the developmental pathways on a molecular level. Generally, we identified zebrafish *cldn5a* as the ortholog of mammalian *CLDN5*, which is essential for maintaining the barrier function of both BBB and BCSFB. In contrast, Cldn5b shows a useless function in the functional formation of zebrafish cerebral barriers, which may illustrate its evolutionary loss in mammals.

## Materials and methods

### Clinical samples and ethics

For analyzing CLDNs expressions in human CNS barriers, clinical samples of lateral ventricle CP and brain parenchyma from a male patient (53 years old) used, who was hospitalized due to left thalamic hemorrhage and was received neuroendoscopic removal of left lateral ventricular hematoma. This study was approved by the Clinical Ethics Committee of the Affiliated Hospital of Guangdong Medical University (Approval number: PJ2021-022).

### Zebrafish strains and maintenance

*cldn5a*^*-/-*^ and *cldn5b*^*-/-*^ were generated in our laboratory as described previously [25]. *cldn5a*^*-/-*^;*cldn5b*^*−/*−  -/- ^ double mutants were obtained by mating *cldn5a*^*+/-*^ and *cldn5b*^*-/-*^ carriers. Transgenic lines of *Tg(kdrl:EGFP)*^*s843*^ and *Tg(coro1α:EGFP)* were used to visualize the vasculature and microglia respectively [26, 27]. All zebrafish were maintained under a 14:10 h light: dark cycle at 28.5 °C by standard guidelines. Handling of zebrafish was performed by Regulations on Laboratory Animal Management of Guangdong Province, China (2010).

### Whole-mount RNA in situ hybridization (WISH)

*cldn5a* and *cldn5b* probes were synthesized as described in our previous study [25]. *clusterin* probe was kindly provided by Dr. Jianfeng Zhou’s group (Ocean University of China). WISH was performed as previously described [28]. Images were taken using a BX53 microscope (Olympus). The area of *clusterin* signal in dCP and mCP was measured by ImageJ software.

### PCR, RT-PCR, and RT-qPCR

For genotyping analysis, embryonic or larval tails were cut and collected in a lysis solution containing protease K For PCR. For RT-PCR, RNA of embryos at different developmental stages were extracted using RNAiso Plus (9109, Takara) following the manufacturer’s protocols. For RT-qPCR, 1000 ng total RNA was first reverse-transcribed using Hifair^®^ III 1st Strand cDNA Synthesis SuperMix for qPCR with gDNA digester plus (11141ES60, YEASEN) on the Eppendorf Mastercycler. PCR and RT-PCR were then performed according to standard protocols with mixtures containing specific primer pairs, DNA/cDNA, and 2× Hieff^®^ PCR Master Mix with Dye (10102ES03, YEASEN). The amplified PCR products was purified with 1.5% agarose gel. For genotyping, after undergoing denaturation and renaturation processes, the DNA products were further cleaved by T7E1 endonuclease (E001L, BEIJING VIEWSOLID BIOTECH), and digestions were separated by 2% agarose gel electrophoresis. For RT-PCR, *β-actin* was used as the loading control and images were captured by ChampGel 7000 (SAGECREATION). RT-qPCR was performed with a mixture of ABI QuantStudio 6 Flex, Hieff^®^ qPCR SYBR Green Master Mix with Low Rox (11202ES08, YEASEN) under the optimized thermocycler conditions recommended by the manufacturer. The specificity of the amplification was evaluated via the melting curve. *gapdh* was used as the internal control. Relative expression of mRNA was calculated by the 2^−ΔΔCt^ method. Sequences of primers for PCR, RT-PCR, and RT-qPCR are listed in Additional file 1: Table S1.

### Histology, immunohistochemistry (IHC), and immunofluorescence (IF)

For IHC or IF staining, fresh mouse brain (C57BL/6, 2 months old) or zebrafish brains were obtained immediately after being sacrificed. The zebrafish and mouse brain, or human brain tissues were then embedded in Tissue-Tek O.C.T. compound (4583, SAKURA) and snap-frozen with dry ice. 8 μm-thick cryosections were performed on a Leica CM1860 UV cryostat and sections were mounted on glass slides. For hematoxylin-eosin (H&E) staining, cryosections were fixed in ice-cold methanol for 5 min and were stained using an H&E staining kit (G1120-3, Solarbio) according to the manufacturer’s protocol. To precisely measure the dilated size of the brain ventricles, we performed serial sections of the whole larval brain. Next, we chose the slides from the consistent/same positions along the axis from arterial to the posterior brain, by referring to the position of the eyeballs (for forebrains) and otic vesicles (for hindbrains), etc. Then, the contour of the ventricles was figured and the area of the midbrain ventricle was measured with ImageJ software. More than 3 sections of each sample were measured, and more than 4 larval brains in each group were analyzed. For IHC and IF, slides were first fixed in acetone on ice for 10 min. After washing and blocking in 5% normal goat serum/PBS for 1 h, the sections were then incubated with primary antibodies diluted in blocking buffer at 4 °C overnight. The next day, slides were incubated with secondary antibodies and DAPI (1:400, D9542, Sigma-Aldrich) for 2 h and subsequently mounted with an anti-fade mounting medium (P0126, Beyotime). All primary and secondary antibodies used are listed in Additional file 1: Table S2. Samples after IHC were imaged under an Olympus BX53 microscope. For the imaging of IF staining, Olympus FV3000 confocal microscope was used. Finally, the mean gray value of Caspase 3 staining was analyzed by ImageJ software. The mean gray values of ZO-1 and Beta-catenin in CP epithelial cells was quantitatively analyzed by ImageJ software.

### Transmission electron microscopy (TEM)

Zebrafish larval brains were fixed in 2.5% glutaraldehyde mixed with 4% PFA in 0.1 M phosphate buffer (PB, pH 7.0). After being washed by 0.1 M PB, samples were post-fixed with 1% osmium tetroxide and dehydrated in graded ethanol. Then, acetone was used to replace ethanol. Tissues were embedded in EPON 812 resin. Ultrathin sections of 60 nm thick were cut and stained with uranyl acetate and lead citrate. Images were captured under HT-7800 TEM (Hitachi, Japan).

### Phylogenetic trees analysis

Phylogenetic trees were generated by the Molecular Evolutionary Genetics Analysis version 11 software (MEGA 11). Protein sequences were aligned by ClustalW methods with default parameters. The Bootstrap method was used to construct the maximum likelihood tree by setting the number of Bootstrap replications as 1000. Bootstrap value was displayed on the branch of the bootstrap consensus tree.

### Movie, microscopy, and imaging

For recording the swimming behavior, zebrafish were raised in normal tanks filled with fish water, and the videos were recorded with a normal camera. For imaging of the larval morphology, zebrafish were anesthetized and mounted in 4% methyl cellulose (M0387, Sigma Aldrich). Images were taken under a Leica M205 FA stereo microscope. The length of the zebrafish body was also measured by ImageJ software. For capturing the anatomical images of the brains, fishes were first sacrificed and fixed in 4% PFA overnight. Then, the whole brain (including the olfactory bulb, fore-, middle- and hindbrain) was dissected and extracted. After taking images of the head and extracting the brain under a stereo-microscope, we calculated the ratio of the brain area to the head area of each larva. Meanwhile, we measured the vertical height (from dorsal to ventral) and the horizontal length (from the anterior olfactory bulb to the end of the posterior hindbrain) of the brain by ImageJ software, and calculated the ratio of vertical/horizontal length of each brain.

For analyzing the distribution of microglia in brains, zebrafish were fixed in 4% PFA overnight. The brains were then collected and embedded in 1% low melting-point agarose (16520100, ThermoFisher Scientific). Images were captured by Olympus FV3000 confocal microscope. The number of microglia was counted and the area of different brain regions was quantified with ImageJ software.

For assessing the pattern of the cerebral vasculature, anesthetized embryos and fixed larval brains were embedded in 1% low melting-point agarose. 3D images were captured by Olympus FV3000 confocal microscope. The diameters of the main cerebral vessels of embryos were further measured by ImageJ software.

### TUNEL assay

Zebrafish were sacrificed and the brains were embedded in the Tissue-Tek O.C.T. compound. Sections 8 μm thick were first fixed in cold acetone on ice for 10 min. After washing, slides were incubated with a TUNEL reaction mix containing the TdT and fluorescein-dUTP for 1 h in dark. Slides were then rinsed several times and stained with DAPI for 1 h. For imaging, tissue samples were mounted in an anti-fade mounting medium and imaged using Olympus FV3000 confocal microscope. The mean gray value of apoptotic signals was evaluated by ImageJ software.

### Assessment of zebrafish BBB and BCSFB integrity

The permeability of brain barriers was evaluated by the diffusion assays using Sulfo-NHS-Biotin (20 mg/ml, A39256, ThermoFisher Scientific) or 10 kDa-Rhodamine B Dextran (2.5 mg/ml, D1824, ThermoFisher Scientific) as tracer or dye. Chemical was injected into the heart ventricle of anesthetized zebrafish using a micromanipulator system. After 10 min of recovery, the heads were cut immediately. For biotin-injected samples, larval heads were embedded in the Tissue-Tek O.C.T. compound directly. Sections 8 μm thick were then fixed in cold acetone on ice for 10 min. After being blocked by H_2_O_2_ for 10 min, sections were incubated with R.T.U (PK-7200, VECTOR LABORATORIES) for 30 min, before staining with 3,3ʹ-diaminobenzidine (DAB-2031, MXB Biotechnologies). The non-injected fish samples were used as negative controls to investigate the endogenous biotin in the zebrafish brain. The methods for signal quantitative analysis are as follows: three regions of brain parenchyma or ventricular side of choroid plexus epithelium are randomly selected in each brain section, and the optical density value was measured by ImagePro plus software to reflect the signal strength of the leaked Sulfo-NHS-Biotin. More than three samples/fishes for each genotype were sectioned and more than three brain slides of each sample were analyzed.

For Rhodamine B Dextran-injected samples, zebrafish heads were fixed in 4% PFA overnight and dehydrated with 30% sucrose in PBS. The next day, samples were embedded in the Tissue-Tek O.C.T. compound and sectioned with a thickness of 8 μm. Then, the sections were stained with DAPI for 2 h and mounted with an anti-fade mounting medium for imaging under Olympus FV3000 confocal microscope. The methods for signal quantitative analysis are as follows: three regions of brain parenchyma or ventricular side of choroid plexus epithelium are randomly selected in each brain section, and the mean gray value of the dye was measured by ImageJ software to reflect the signal strength of the leaked dye. More than three samples/fishes for each genotype were sectioned and more than three brain slides of each sample were analyzed.

### Cell culture

Human brain microvascular ECs (HBMEC), mouse brain microvascular ECs (bEnd.3), and Madin-Darby canine kidney cells (MDCK-I) were cultured in DMEM containing 10% fetal bovine serum at 37 °C in a 5% CO_2_ incubator. The HBMEC *CLDN5*^MUT^ and bEnd.3 *CLDN5*^MUT^ cell lines were generated using the CRISPR/Cas9 strategy as previously described [25]. For cell transfection, MDCK-I cells were transfected with *pIRES2-C1-CLDN5* (Human) plasmid using Lipofectamine™ 2000 (11668019, ThermoFisher Scientific) following the manufacturer’s instructions. Stable cell lines were obtained from monoclonal cells after selection with G418 (800 μg/ml). The expression of *CLDN5* in transfected MDCK-I cells was further analyzed by IF assay.

### Assessment of permeability of barrier in vitro

The values of trans-epithelial/endothelial electrical resistance (TEER) and paracellular tightness were measured to evaluate the permeability of the barriers in vitro. For TEER measurement, HBMEC cells and MDCK-I cells were seeded onto the transwell filter inserts (3470, Corning) respectively. Monolayer electrical resistance was measured by EVOM2 (World Precision Instruments). For paracellular permeability analysis using 10 kDa-FITC dextran (0.5 mg/ml, D1821, ThermoFisher Scientific), the dye was applied to the monolayer cells. After culturing for 1 h, cell culture in the lower chamber was collected and the leakiness of the endothelial monolayer was evaluated by measuring the fluorescent signals in the medium using a plate reader (MK3, ThermoFisher Scientific).

### Cell proliferation and apoptosis assay

Cell proliferation of HBMEC cells and bEnd.3 cells was detected using Cell Counting Kit-8 (JE603, Japan Dojingdo Molecular Technologies) following the manufacturer's instructions. Cell apoptosis was evaluated with Annexin V-FITC Apoptosis Detection Kit (C1062M, Beyotime Biotechnology) according to the instructions.

### Statistical analysis

All the experiments were repeated at least three times. Statistical analyses were performed using GraphPad Prism 8.0 software. The results were presented as mean ± SEM. Unpaired two-tailed Student’s *t* test was used for comparisons between two groups. Two-way ANOVA analysis was used in the TEER experiments. P value < 0.05 was considered to be statistically significant, marking with *P < 0.05, **P < 0.01, ***P < 0.005, ****P < 0.001.

## Results

### Expression of Claudins in mammalian brains

To identify the exact expression pattern of Cldn5a and Cldn5b in zebrafish CNS, and to explore the mammalian orthology of zebrafish *cldn5a* and *cldn5b*, we first investigated the expressions of several typical CLDN family members in human and mouse brain. As shown in Additional file 1: Fig. S1A and B, CLDN5 was detected strongly expressed in human or mouse cerebrovascular ECs but not in CP epithelium, while CLDN1 and CLDN3 were mainly expressed in mammalian CP epithelium. In addition, CLDN1 and CLDN3 showed weak expression in the ECs of cerebral vessels (Additional file 1: Fig. S1A, B), probably due to the cross-reactivity of antibodies as previously reported [19, 29]. In contrast, CLDN7 was not detected in mammalian cerebral ECs or epithelial cells of CP (Additional file 1: Fig. S1A), consistent with the findings that it is mainly found in the stomach, lung, intestine, bladder, and kidney [30]. Above expression patterns of mammalian CLDNs and the analyzed phylogenetic tree of these CLDNs in human, mouse, and zebrafish (Additional file 1: Fig. S2) suggests zebrafish *cldn5s* as the orthologs of mammalian *CLDN5*.

### Spatiotemporal expression of the Cldn5a and Cldn5b during zebrafish development

During embryonic development, zebrafish *cldn5a* mRNA was detectable as early as 6 hpf with a gradual increase of its expression level to 12 hpf (Additional file 1: Fig. S3A). WISH analyses of *cldn5a* indicated a strong expression in neuroepithelium at 30 hpf (Additional file 1: Fig. S3B) and a remarkable expression in both diencephalic choroid plexus (dCP) and myelencephalic choroid plexus (mCP) at 5 dpf, as indicated by the CP specific marker of *clusterin* (Additional file 1: Fig. S3C) [31]. To show the precise expression of Cldn5a protein in 5 dpf embryos, IHC against Cldn5 followed by a cross-section through the brain region revealed that Cldn5 colocalized with *clusterin* mRNA signals specifically in the epithelial cells of dCP and mCP (Additional file 1: Fig. S3D). In parallel, we explored the expression of *cldn5b* in zebrafish embryos. RT-PCR analysis indicated that the expression of *cldn5b* was detectable already at the one-cell stage and gradually decreased to the lowest level at 6 hpf while subsequently increased to 2 dpf (Additional file 1: Fig. S3A), suggesting a maternal-zygotic expression of *cldn5b* during early embryonic development. WISH with embryos showed that *cldn5b* was expressed in cerebral vasculatures at 30 hpf (Additional file 1: Fig. S3B). Additionally, the expression of *cldn5a* and *cldn5b* in different organs of adult zebrafish was also analyzed and the RT-PCR revealed that *cldn5b* was ubiquitously expressed in zebrafish tissues including brain, heart, and liver while *cldn5a* was mainly detectable in the adult brain (Additional file 1: Fig. S3E).

**Fig. 1:**
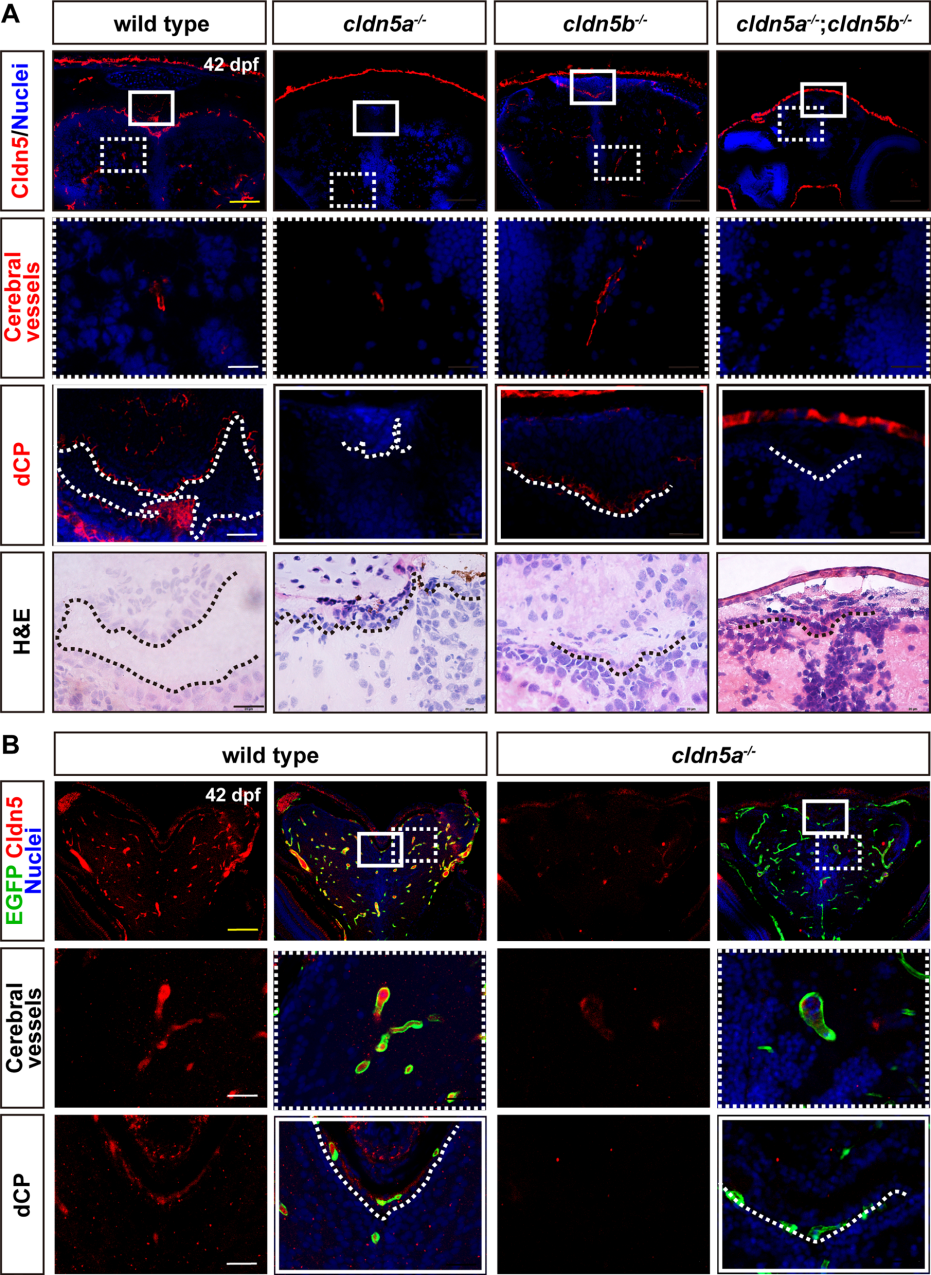
Expression patterns of Cldn5a and Cldn5b in zebrafish brain. The brain sections of 42 dpf-old zebrafish were stained by an anti-pan Cldn5 antibody. **A** In wild types, Cldn5s are not only expressed in cerebrovascular ECs but also the dCP epithelium. In *cldn5a*^*-/-*^, Cldn5b expresses only in a few cerebrovascular ECs but not in dCP. In *cldn5b*^*-/-*^, Cldn5a is strongly expressed in both cerebrovascular ECs and dCP. In *cldn5a*^*-/-*^;*cldn5b*^*-/-*^, no specific signals could be detected in the brain. Serial sections were stained with HE to show the histology of dCP. **B** *Tg(kdrl:EGFP)* zebrafish was introduced to help to determine the endothelial Cldn5 expression. In *cldn5a*^*-/-*^, Cldn5b is only expressed within a few cerebral vessels but not in dCP epithelium. White rectangles or dashed rectangles indicate the enlarged regions of dCP, or cerebral vessels respectively which are shown in the lower panels with high magnification. White dotted lines or black dotted lines show the continuous CP epithelium. n = 3 fishes analyzed per group. Scale bars, 100 μm in yellow or 20 μm in black or white

Since the current available CLDN5 antibody is a pan-antibody that recognizes both zebrafish Cldn5s, to further determine the detailed expression of Cldn5a and Cldn5b, the specific *cldn5a* and *cldn5b* mutants were generated in our lab [25]. Firstly, the specific expression of endogenous *cldn5s* in the mutated lines was detected by RT-qPCR at the developmental stages of 5 dpf and 20 dpf respectively. The results indicated that *cldn5a* or *cldn5b* was efficiently knocked out in *cldn5a*^*-/-*^ or *cldn5b*^*-/-*^ , and both *cldn5s* were lost in *cldn5a*^*-/-*^;*cldn5b*^*-/-*^ double mutant at the embryonic stage of 5 dpf (Additional file 1: Fig. S3F, G). These results ensure that there is no genetic compensation effect between *cldn5a* and *cldn5b* in the corresponding *cldn5* mutants. Next, the specific expression of Cldn5a and Cldn5b in the brains was analyzed in juvenile zebrafish. As shown in Fig. 1A and Additional file 1: Fig. S4A, in wild types, the signal of Cldn5 was detected not only in cerebral vessels but also in dCP and mCP. In *cldn5a*^*-/-*^, Cldn5b expressed weakly in cerebral ECs but not in dCP and mCP, while in *cldn5b*^*-/-*^, besides the strong expression in cerebral vessels, Cldn5a was also expressed in dCP and mCP (Fig. 1A; Additional file 1: Fig. S4A). As expected, in *cldn5a*^*-/-*^;*cldn5b*^*-/-*^ double mutant, there was no expression of Cldn5 in cerebral vessels nor CPs. Moreover, to confirm the expression of Cldn5a in CP epithelium more specifically, transgenic *Tg(kdrl:EGFP)* line with green fluorescent protein-labeled ECs was applied. As shown in Fig. 1B and Additional file 1: Fig. S4B, Cldn5a in CP epithelium was lost in *cldn5a*^*-/-*^ in comparison to that in wild types, leaving only weak expression of Cldn5b in partial cerebral ECs, which was consistent with above findings in Fig. 1A and Additional file 1: Fig. S4A. In general, these results identified a specific expression of Cldn5a in both cerebral ECs and CP epithelial cells, while Cldn5b only expressed weakly in cerebrovascular ECs.

### *cldn5a* mutant shows fatal brain edema

**Fig. 2:**
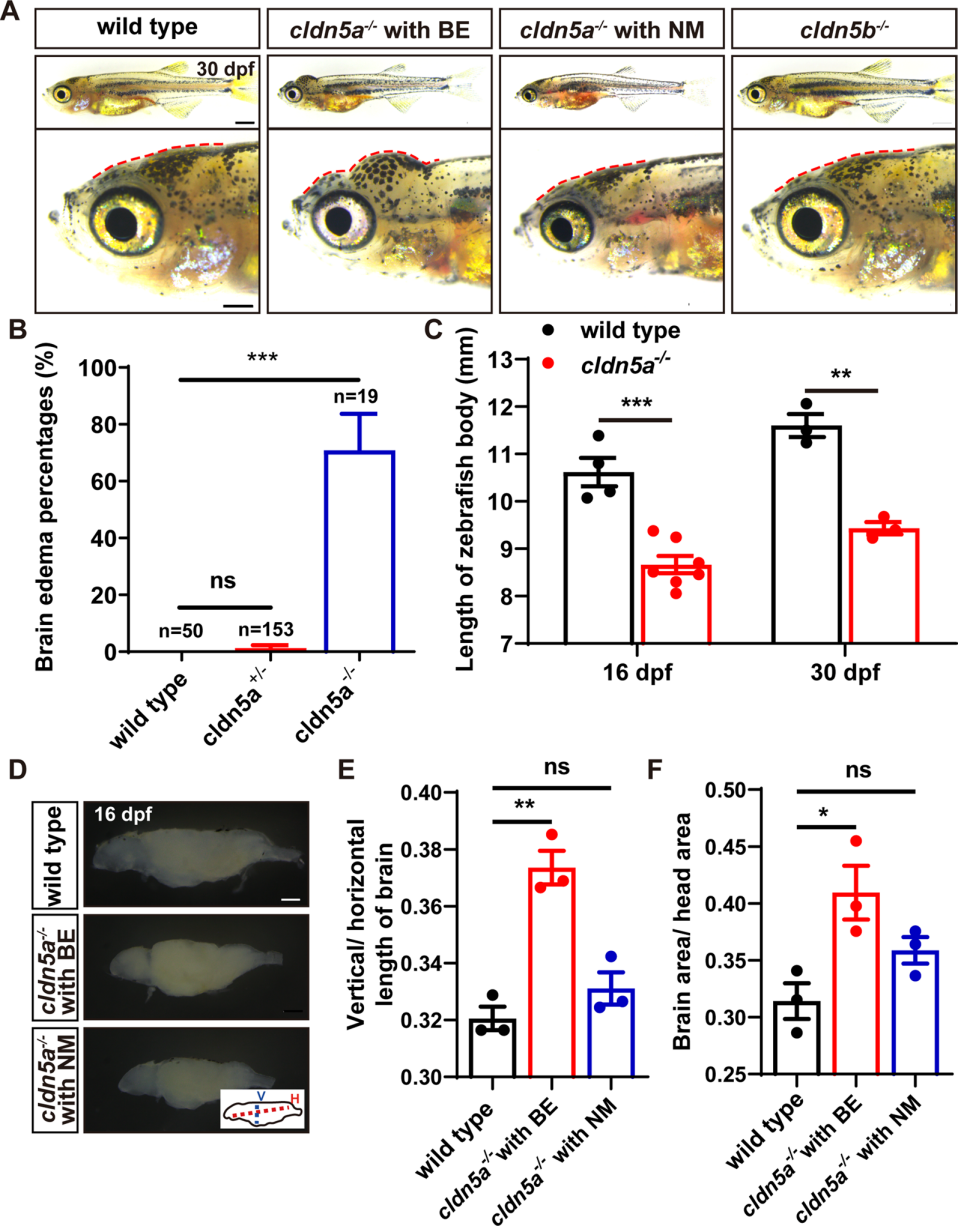
Fetal brain edema (BE) in *cldn5a*^*-/-*^ zebrafish. **A**, **B** Around 70% of *cldn5a*^*-/-*^ mutated zebrafish larvae show an obvious morphology with a tiny body and serious swollen head (BE), and the rest of the homozygous mutants have normal morphology (NM) but a tiny body. *cldn5b*^*-/-*^ shows normal and healthy morphology as wild types. Enlarged figures of the head regions are shown in the lower panel. Red dotted lines figure out the profile of the skull. **C** *cldn5a*^*-/-*^ larvae showed a tiny body at both 16 and 30 dpf. **D**-**F** Dissected brain tissues of 16 dpf-old larvae from wild types, *cldn5a*^*-/-*^ with BE, and *cldn5a*^*-/-*^ with NM brains. The ratio of vertical/horizontal length of the whole-brain was analyzed. The ratios of the brain area to head area was calculated and compared. V, vertical. H, horizontal. n > 3 brains analyzed per group. Scale bars: 1 mm. Data are presented as mean ± SEM. *P < 0.05, ***P < 0.005, ns means no significance

Our previous study has reported that *cldn5a*^*-/-*^ mutated embryos show a lethal phenotype of a non-expanded dorsal aorta with a penetrance of 30% to 40% at around 24 hpf stage, due to the loss of Cldn5a-generated EC adhesive force [25]. Here, we subsequent tracked the fate of the survived *cldn5a*^*-/-*^ with well-formed vasculatures and we found that at the stage of 20-30 dpf, around 74% of the *cldn5a*^*-/-*^ larvae (14 out of 19 larvae) died with severe brain edema (BE) (Fig. 2A, B). In contrast, the *cldn5a*^*+/-*^ or *cldn5b*^*-/-*^ showed normal morphology without any defects, like the wild type larvae (Fig. 2A, B). In addition, *cldn5a*^*-/-*^ showed tiny bodies (Fig. 2C). Next, to ask whether the swollen head phenotype of *cldn5a*^*-/-*^ was due to hydrocephalus or skin oedema, we dissected the head and extract the brain of the larvae. As a result, we found there was no obvious cerebrospinal fluid effusion but an obvious brain swelling observed was observed inside the head of *cldn5a*^*-/-*^ with BE (Fig. 2D). By comparing the vertical and horizontal length of the whole brain, we found that due to BE, *cldn5a*^*-/-*^ with BE showed a higher vertical to horizontal length ratio than that of the brains of the wild types or *cldn5a*^*-/-*^ with NM at 16 dpf (Fig. 2E). Meanwhile, *cldn5a*^*-/-*^ with BE showed a higher brain to head ratio of size than that of the wild types or *cldn5a*^*-/-*^ with NM at 16 dpf (Fig. 2F). Moreover, the behavior analysis indicated that in comparison to wild type, *cldn5a*^*-/-*^ exhibited defective swimming behaviors at both 20 and 42 dpf stages, such as pirouette, and rotating movements (Additional file 2: Movie S1 and Additional file 3: Movie S2).

### Whole-brain oedema, ventricular dilatation, and cerebral hernia in *cldn5a*^*-/-*^ with BE

**Fig. 3:**
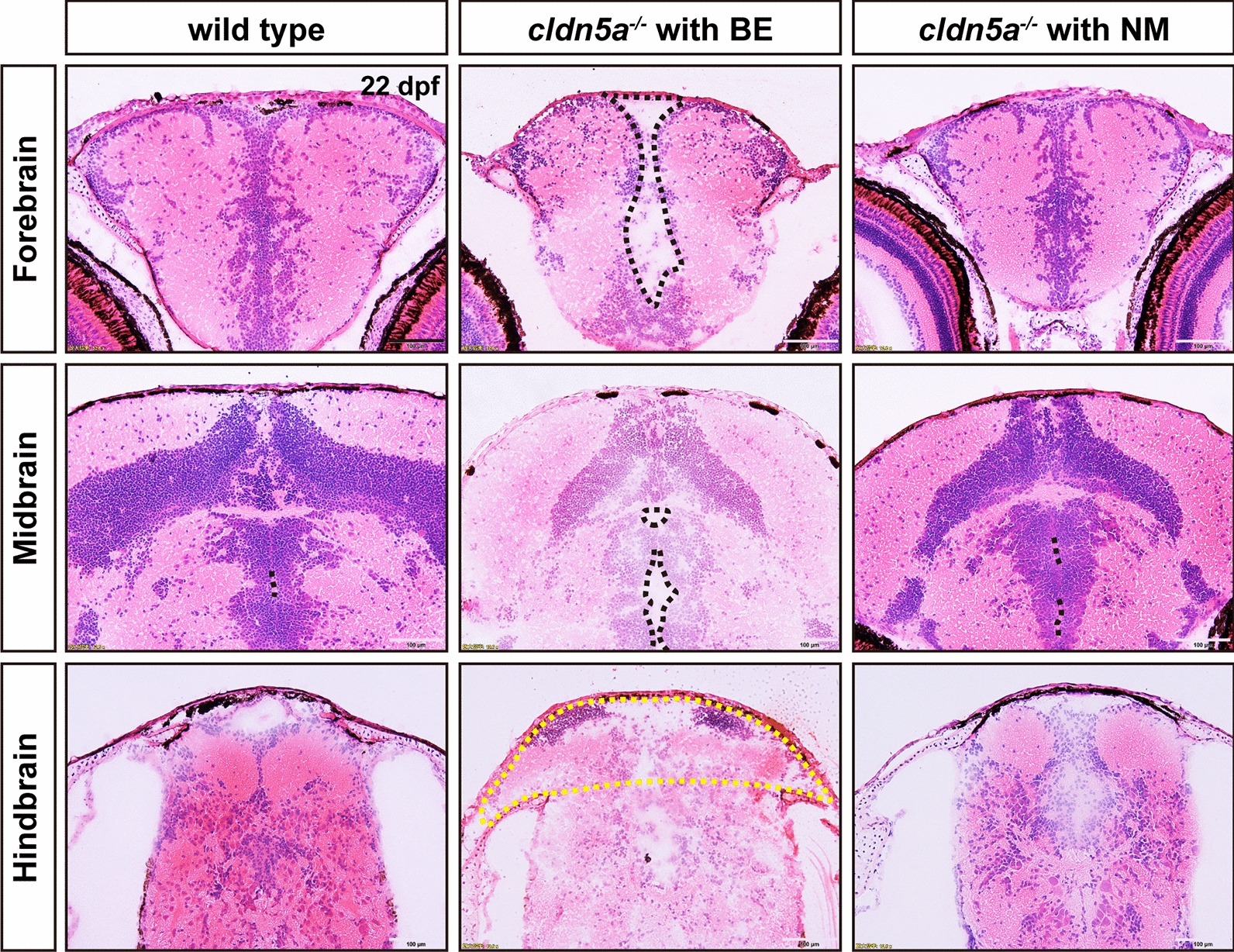
Whole-brain oedema, ventricular dilatation, and cerebral hernia in *cldn5a*^*-/-*^ with brain edema (BE). Histology of and HE staining on zebrafish brain sections at 22 dpf. In the forebrain and midbrain, *cldn5a*^*-/-*^ with BE had a round outline and exhibited serious brain parenchymal edema and exaggerated ventricular dilatation. A yellow dot line labels the brain hernia in *cldn5a*^*-/-*^ with BE. Black dot lines describe the brain ventricles. n > 3 brains analyzed per group. Scale bars: 100 μm

To better characterize the BE phenotype observed *cldn5a*^*-/-*^, H&E staining following cryosection across the larval brains were performed to detect the histological changes in zebrafish at 22 dpf. The results showed that compared to wild types and *cldn5a*^*-/-*^ with NM, *cldn5a*^*-/-*^ with BE exhibited severer oedema in the whole-brain but without cerebral hemorrhage, and the encephalic parenchyma was loosed in structure and filled with a large amount of interstitial fluid (Fig. 3). *cldn5a*^*-/-*^ with BE displayed dilated ventricles in all the fore-, mid-, and hindbrains (Fig. 3). We further quantitatively analyzed the area of the midbrain ventricle. As shown in Additional file 1: Fig. S5, the ventricular area of the midbrain of *cldn5a*^*-/-*^ with BE increased significantly. There was no significant difference in the ventricular area of the midbrain between *cldn5a*^*-/-*^ with NM and wild type. Moreover, a severe cerebral hernia was observed in the hindbrains of *cldn5a*^*-/-*^ with BE (yellow circle in Fig. 3). Compared with the wild types, there was no obvious alteration in the brains of *cldn5a*^*-/-*^ with NM, except for slight dilatations in the hindbrain ventricle (black dash lines in Fig. 3), suggesting the edema of *cldn5a*^*-/-*^ brain might form in a gradual pathological process.

### Cerebral inflammation and neuronal apoptosis in *cldn5a*^*-/-*^ with BE

Cerebral inflammation and apoptosis are usually concomitant and interactive with BE during the pathophysiological processes of various CNS diseases [32–34]. Hence, to detect the cerebral inflammation in the heads of *cldn5a*^*-/-*^ with BE, we investigated the mRNA expression levels of the pro- and anti-inflammatory cytokines by RT-qPCR assay. As shown in Additional file 1: Fig. S6A, *il-8* and *il-1b* coding for pro-inflammatory factors, was highly expressed in *cldn5a*^*-/-*^ brains with BE. In contrast, compared with that in wild type siblings, the expression *il-8* and *il-1b* showed no difference in *cldn5a*^*-/-*^ with NM (Additional file 1: Fig. S6A).

As the resident macrophages of the CNS, microglia orchestrate neuroinflammation by secreting pro-inflammatory cytokines like IL-1β and IL-8 [35, 36]. Therefore, we also applied *Tg(coro1α:EGFP)* transgenic zebrafish in which GFP labels macrophages to investigate the distributions of microglia in *cldn5a*^*-/-*^ brain. Unexpectedly, it was observed that the average number of microglia in the midbrain and hindbrain of *cldn5a*^*-/-*^ with BE showed no significant difference compared with that in wild-type siblings, except for a slight decrease in the forebrain of *cldn5a*^*-/-*^ with BE (Additional file 1: Fig. S6B, C). The distribution of microglia in dCP and mCP was also analyzed and it was found that the number of microglia in CP of *cldn5a*^*-/-*^ with BE was much less than that in wild types (Additional file 1: Fig. S6D, E).

Since an unexpected smaller number of microglia in CP of *cldn5a*^*-/-*^ with BE was observed, we next questioned whether this was due to the cell apoptosis caused by edema in the whole cerebral parenchyma. To this end, cross-sections through the brain region followed by cell apoptosis analyses were performed. The results of both the TUNEL assay (Additional file 1: Fig. S7A, B) and anti-Caspase 3 stainings (Additional file 1: Fig. S7C, D) revealed that in addition to cerebral inflammation, a large amount of apoptosis occurred in the brains of *cldn5a*^*-/-*^ with BE. However, in *cldn5a*^*-/-*^ without BE yet, there was no obvious apoptosis detected in neuronal cells (Additional file 1: Fig. S7A-D). Furthermore, ultrastructural analysis of the brain sections provided direct evidence of neuronal apoptosis and cell necrosis in zebrafish larval brains of *cldn5a*^*-/-*^ with BE (Additional file 1: Fig. S7E).

### Cldn5a is required for both BBB and BSCFB in zebrafish larvae

As a canonical role, mammalian CLDN5 functions in maintaining the permeability of CNS barriers [17]. Since we have observed strong expressions of zebrafish Cldn5a in both cerebrovascular ECs and CP epithelial cells, we next asked about the functional integrity of both zebrafish BBB and BCSFB in *cldn5a* mutant respectively. To this end, two tracers with different molecular sizes, sulfo-NHS-biotin (443 Da) and rhodamine dextran (10 kDa), were micro-injected into the circulation systems of 20-22 dpf larvae. Since *cldn5a*^*-/-*^ with BE showed serious destructive brain histology (Fig. 3) and severe neuronal apoptosis (Additional file 1: Fig. S6), and was not suitable for the evaluation of cerebral barriers technically, we therefore applied *cldn5a*^*-/-*^ brain with NM to determine the barrier function of BBB and BCSFB directly.

**Fig. 4:**
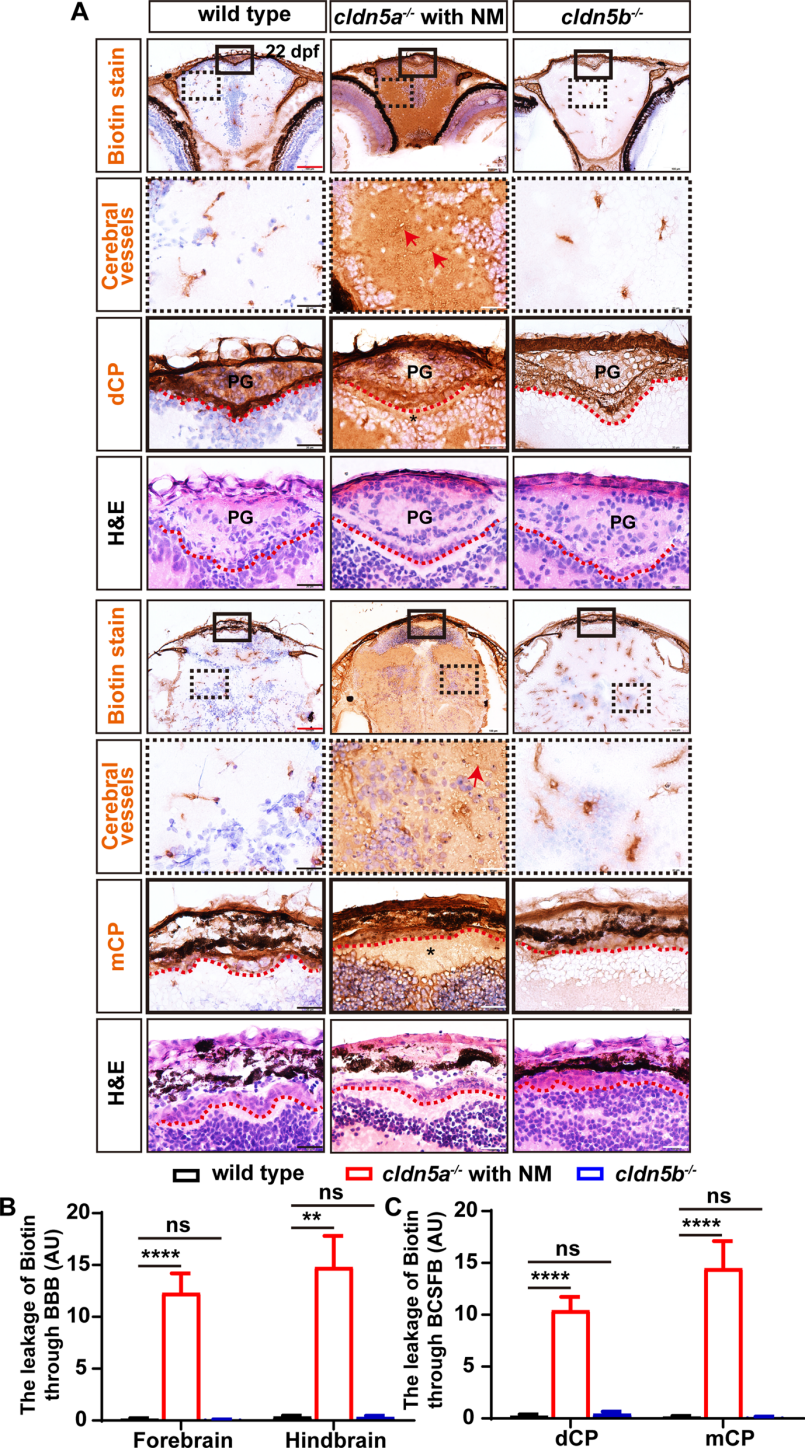
The leakage of sulfo-NHS-biotin through BBB and BCSFB in *cldn5a*^*-/-*^. **A** Sulfo-NHS-biotin (443 Da) was injected into the circulation system to assess the tightness of BBB and BCSFB in 22 dpf-old zebrafish larvae. In wild-type biotin signals are distributed in cerebral vessels, pineal gland (PG), and inside the CPs (both dCP and mCP), but not in brain parenchyma and ventricle. In *cldn5a*^*-/-*^ with NM, biotin was permeated through the cerebrovasculatures (red arrows) into the whole brain. In the brains of *cldn5b*^*-/-*^, biotin stays in the cerebral vessels, PG, and inside the CPs. Serial sections were stained with HE to show the histology of CPs. Black dashed rectangles and solid rectangles indicate the enlarged regions of cerebral vessels and CPs respectively which are shown in the lower panels with high magnification. Red dot lines describe the linear CP epithelial cells. Black asterisks indicated the brain ventricles beneath the CPs with leaky biotin. Scale bars: 100 μm in red and 20 μm in black. **B** Quantification of the biotin signal strength of the biotin leaks through BBB into brain parenchyma. **C** Quantification of the signal strength of the leaked biotin through BCSFB. n > 3 fishes analyzed per group. Data are represented as mean ± SEM; **P < 0.01, ****P < 0.001. ns means no significance

Firstly, the interference of endogenous biotin in larval brains was excluded in the sulfo-NHS-biotin diffusion assay (Additional file 1: Fig. S8). In wild type, biotin was detected only being restricted in cerebral vessels, pineal gland, and CP (including dCP and mCP), while there was no biotin signal detected in brain parenchyma or brain ventricles (Fig. 4A). In particular, we found that the pineal gland was also stained, probably due to the fenestrated vessels inside the CP through which biotin diffused. Compared to biotin distributions in wild type, it was found in the brain of *cldn5a*^*-/-*^ with NM, biotin permeated from the cerebral vessels into brain parenchyma severely (red arrows in Fig. 4A), and the diencephalic and myelencephalic ventricles were filled with biotin (black asterisks in Fig. 4A), indicating leakage of both BBB and BCSFB in *cldn5a*^*-/-*^ larvae. Additionally, since the above results have revealed an expression of zebrafish Cldn5b in the brain capillaries, we analyzed the integrity of BBB or BCSFB in *cldn5b*^*-/-*^ in parallel. The results showed that biotin could not diffuse out the cerebral vessels, indicating a non-functional role of Cldn5b in the maintenance of BBB (Fig. 4A). We further evaluated the leakage of biotin through BBB and BCSFB in *cldn5a*^*-/-*^ and *cldn5b*^*-/-*^. As shown in Fig. 4B and C, loss of Cldn5a in 22 dpf-old larvae caused leaky BBB and BCSFB comparing to their tightness in wild-type sibilings.

**Fig. 5:**
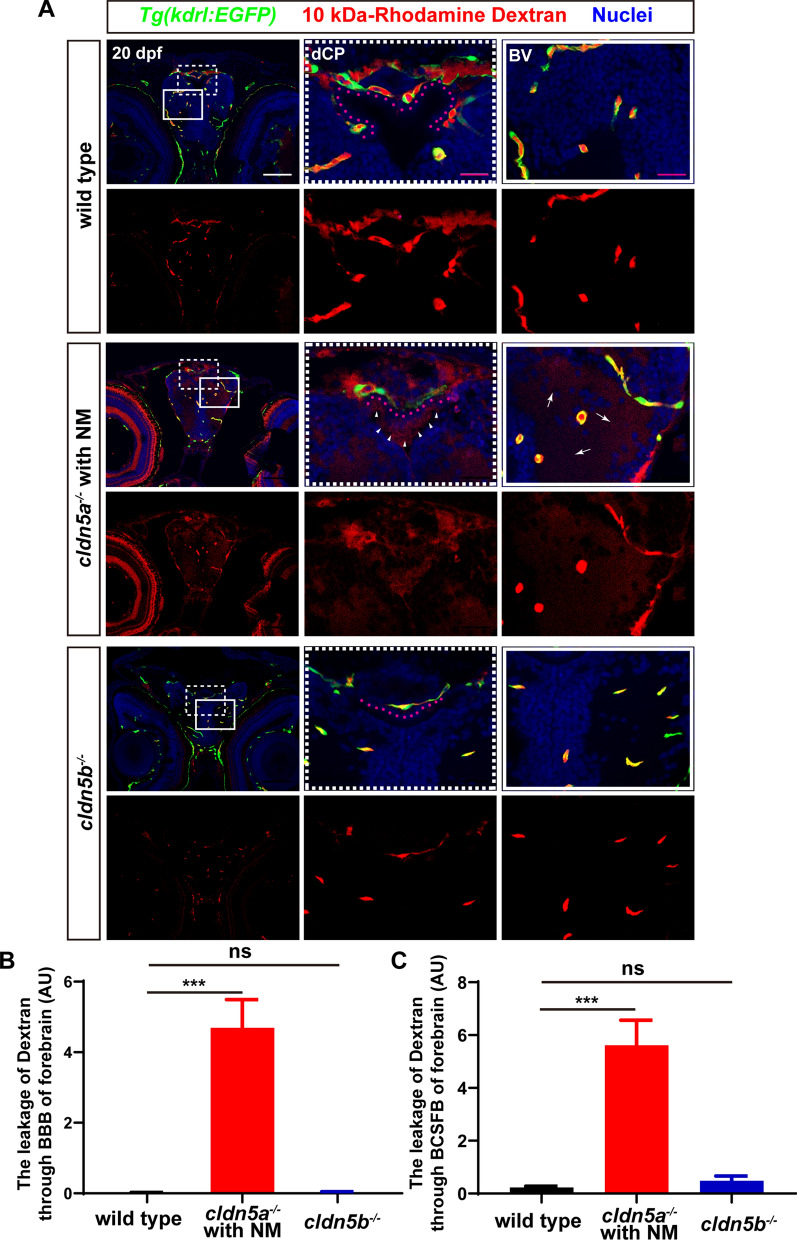
The leakage of 10 kDa-rhodamine dextran through BBB and BCSFB in the forebrain of *cldn5a*^*-/-*^. **A** In the forebrain sections of *cldn5a*^*-/-*^ with NM, rhodamine dextran penetrates through brain capillaries and strongly accumulated in brain parenchyma (white arrows). Meanwhile, rhodamine dextran was found to leak through the paracellular space of the dCP epithelium (pink dashline) and fullfill the forebrain ventricle (white arrowheads). White dashed rectangles and solid rectangles indicate the enlarged regions of dCP and cerebral vessels respectively which are shown on the right side with high magnification. Pink dot lines label the position of the dCP epithelial cells. Scale bars: 100 μm in white and 20 μm in pink. **B** Quantification of the signal strength of the dye leaked through BBB into the forebrain parenchyma. **C** Quantification of the signal strength of the dye leaked through BCSFB into the forebrain ventricle. *Tg(kdrl:EGFP)* line was applied to label the cerebral vessels. BV, blood vessel. n > 3 fishes analyzed per group. Data are represented as mean ± SEM; ***P < 0.005. ns means no significance

**Fig. 6:**
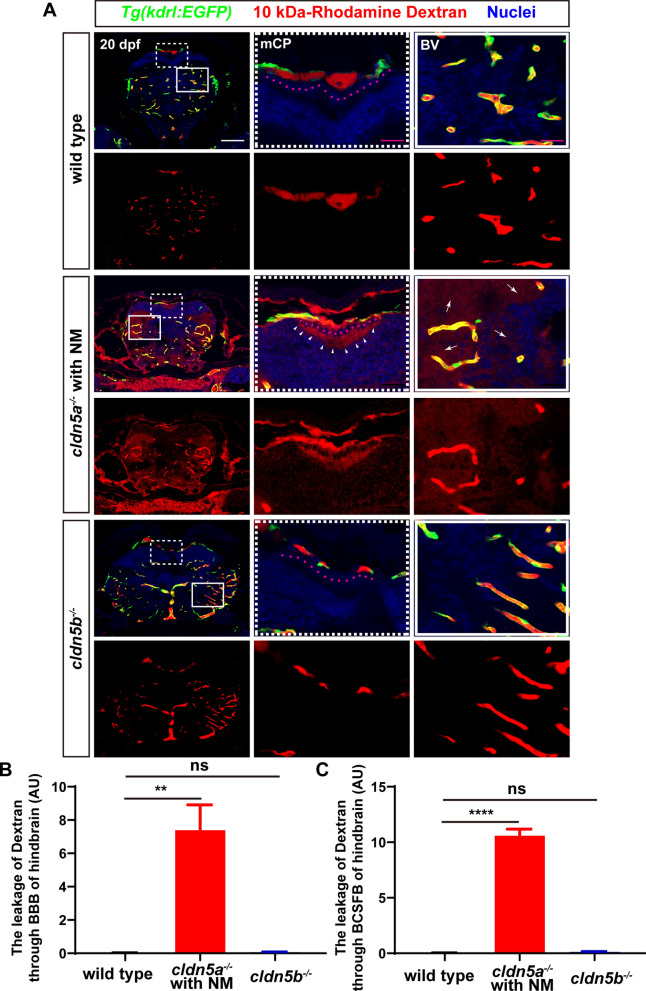
The leakage of 10 kDa-rhodamine dextran through BBB and BCSFB in the hindbrain of *cldn5a*^*-/-*^. **A** In the hindbrain sections of *cldn5a*^*-/-*^ with NM, rhodamine dextran penetrates through brain capillaries and strongly accumulated in brain parenchyma (white arrows). Meanwhile, rhodamine dextran was found to leak through the paracellular space of the mCP epithelium (pink dashline) and fullfill the hindbrain ventricle (white arrowheads). White dashed rectangles and solid rectangles indicate the enlarged regions of mCP and cerebral vessels respectively which are shown on the right side with high magnification. Pink dot lines label the position of the mCP epithelial cells. Scale bars: 100 μm in white and 20 μm in pink. **B** Quantification of the signal strength of the dye leaked through BBB into the hindbrain parenchyma. **C** Quantification of the signal strength of the dye leaked through BCSFB into the hindbrain ventricle. *Tg(kdrl:EGFP)* line was applied to label the cerebral vessels. BV, blood vessel. n > 3 fishes analyzed per group. Data are represented as mean ± SEM; ***P < 0.005. ns means no significance

To further study the integrity of BBB and BCSFB barrier in *cldn5a*^*-/-*^ and *cldn5b*^*-/-*^, a bigger size tracer of 10 kDa-rhodamine dextran was microinjected into the larval vessels again. To this end, *Tg(kdrl:EGFP)* line was applied to label the cerebral vessels. As shown in Figs. 5 and 6, in *cldn5a*^*-/-*^ brains, rhodamine dextran leaked through the BBB into the brain parenchyma closed to cerebral vessels (white arrows in Figs. 5A and 6A), or passed through the BCSFB and accumulated to the apical surface of CP epithelium (white arrowheads in Figs. 5A and 6A) severely. The quantitative analyses by comparing the signal strengths of leaked fluorescent dye in *cldn5a*^*-/-*^, and wild type indicated a strong diffusion of rhodamine dextran through BBB and BCSFB in *cldn5a*^*-/-*^ (Figs. 5B, C and 6B, C). In contrast, the BBB function of *cldn5b*^*-/-*^ was not altered due to the loss of Cldn5b.

The above findings revealed that zebrafish Cldn5a but not Cldn5b is necessary for maintaining the tightness of BBB, and moreover, Cldn5a contributes to BCSFB function. We also explored in vitro evidence to support the findings of the essential role of CLDN5 in endothelial and epithelial barriers. By knocking out *CLDN5* in human mice brain microvascular ECs (HBMEC) or mice brain ECs (bEnd.3) respectively, the endothelial tightness was evaluated by TEER assay in HBMEC cells and permeability assay in bEnd.3 cells. The results revealed that loss of CLDN5 increased the monolayer EC permeability largely (Additional file 1: Fig. S9A-D). Meanwhile, human *CLDN5* was transfected into MDCK-I cells to express exogenous CLDN5 stably (Additional file 1: Fig. S9E), and as expected, the tightness of the MDCK-I cell monolayer was enhanced by the expression of exogenous *CLDN5* as reflected by TEER measurement (Additional file 1: Fig. S9F).

Altogether, the above in vivo and in vitro data suggest that CLDN5 plays important role in maintaining both the endothelial barrier of BBB and the epithelial barrier of BCSFB.

### The development of cerebral vessels and choroids plexus is independent of Cldn5a

**Fig. 7:**
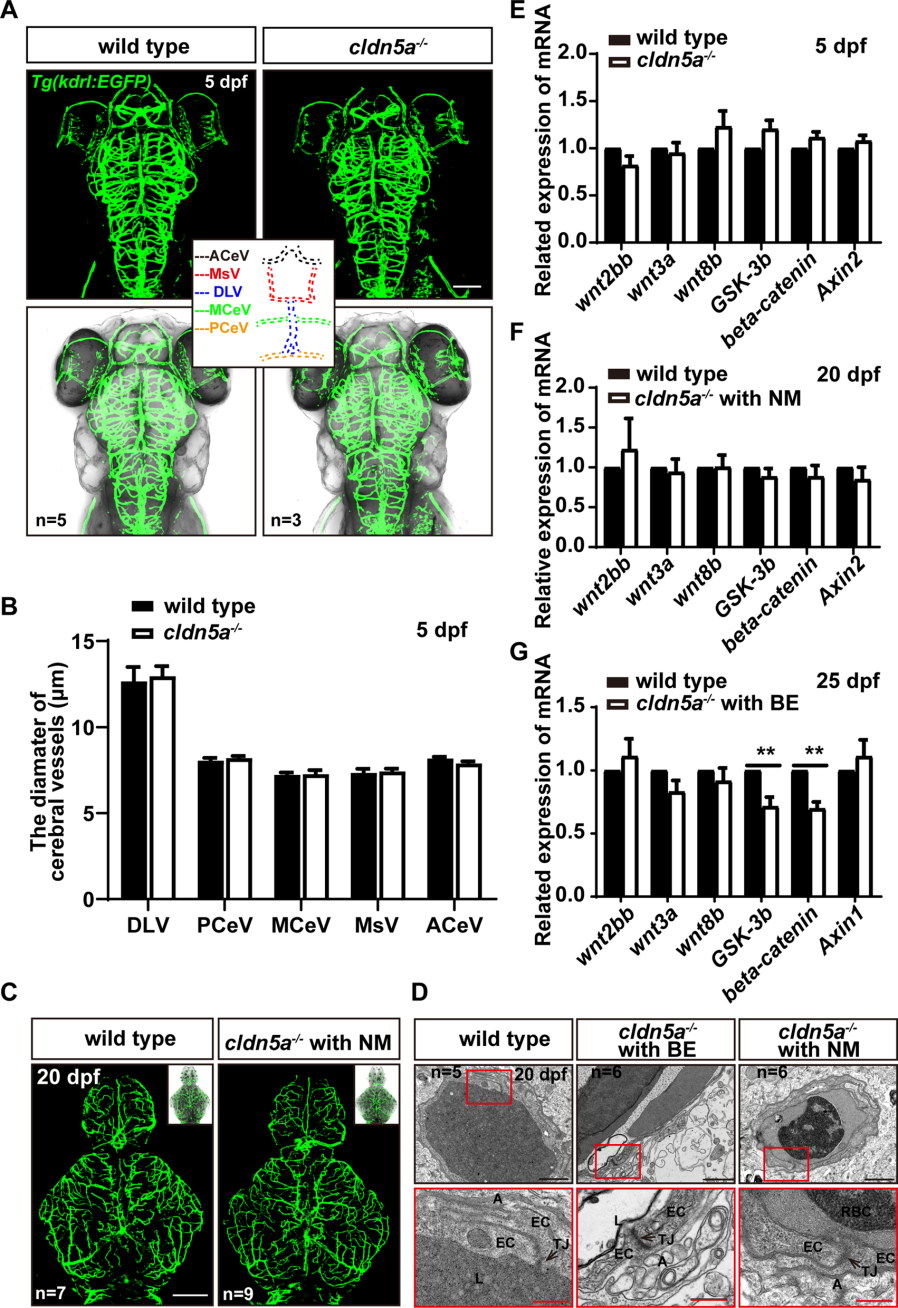
The formation of cerebral vessels was not affected in *cldn5a*^*-/-*^. **A**-**C** The endothelial transgenic line of *Tg(kdrl:EGFP)* was applied to assess the vasculogenesis and angiogenesis of cerebral vessels in *cldn5a*^*-/-*^. The patterns and the vascular density of cerebral vessels were not altered in *cldn5a* at 5 dpf (**A**) or 20 dpf (**C**). The diameters of several main cerebral vessels including anterior cerebral vein (ACeV), mesencephalic vein (MsV), dorsal longitudinal vein (DLV), middle cerebral vein (MCeV), and posterior cerebral vein (PCeV) show no discrepancy between wild types and *cldn5a*^*-/-*^ (**B**). **D** Ultrastructure of cerebral vascular ECs and TJ strands in *cldn5a*^*-/-*^ and wild types at 20 dpf. The morphology of cerebral vascular ECs and the integrity of TJs in *cldn5a* mutants is normal as in wild types. The regions labeled by red rectangles show EC junctions which are presented in the lower panel with high magnification. Black arrows indicate paracellular TJs. EC, endothelial cell; L, vascular lumen; A, abluminal side; RBC, red blood cell. n > 5 fishes analyzed per group. Scale bars: 1 μm in black and 500 nm in red. **E**-**G** The expression level of genes coding Wnt pathway factors related to vasculogenesis or angiogenesis. No significant discrepancy was detected in the expression of genes related to the Wnt pathway at 5 dpf embryos or 20 dpf larvae between wild type and *cldn5a*^*-/-*^. A decrease of *gsk3b* and *beta-catenin* was detected in *cldn5a*^*-/-*^ with BE to that in wild types at 25 dpf larvae. Each experiment was repeated more than three times. Data are shown as mean ± SEM. *P < 0.05, **P < 0.01

In mice, loss of CLDN5 causes a loosened BBB while does not alter the vasculogenesis of cerebral vessels [19]. Herein, we next sought to identify whether the development of cerebral vessels or formation of CP in *cldn5a*^*-/-*^ was affected due to the loss of *cldn5a*. To this end, the patterns including the vascular density and diameter of main cerebrovasculatures of 5 dpf embryos were investigated first. The results showed that loss of Cldn5a did not alter the cerebrovascular density and diameters at 5 dpf (Fig. 7A, B). Meanwhile, the cerebral vascular pattern was also investigated at a later stage of 20 dpf. The results revealed that there was no significant difference of the cerebral vascular density and morphology between *cldn5a*^*-/-*^ and wild-type siblings (Fig. 7C). Furthermore, ultrastructural analysis of the brain capillaries revealed that the TJ strands were still maintained in the absence of Cldn5a (Fig. 7D). However, the structures of brain parenchyma and cerebrovasculature were severely affected in *cldn5a*^*-/-*^ with BE, and the basement membrane of blood vessels was lost (Fig. 7D). To additionally clarify the role and necessity of CLDN5 on the growth of the EC layer, we analyzed the growth properties of *CLDN5*^WT^ and *CLDN5*^MUT^ cell lines of HBMEC and bEnd.3 respectively. As expected, the proliferation rate (Additional file 1: Fig. S10A, B) or the cell apoptosis of both HBMEC cells and bEnd.3 cells was not altered due to the loss of CLDN5 (Additional file 1: Fig. S10C-F). These are consistent with the findings in *CLDN5* knocked out mice where cerebral vessel density and endothelial TJ were not altered.

Next, to explore whether the molecular pathways required for vasculogenesis was affected by the loss of Cldn5a, the gene expression levels of classic Wnt pathway factors were analyzed [37]. As shown in Fig. 7E and F, the expression levels of Wnt pathway-related genes were not affected in *cldn5a*^*-/-*^ during either the early embryonic stage of 5 dpf nor the larval stage of 20 hpf. However, in *cldn5a*^*-/-*^ with severe BE at 25 dpf, it was found a decrease of the expression level of *gsk3b* and *beta-catenin*, probably due to the pathophysiological processes in the brain such as neuronal apoptosis (Fig. 7G).

**Fig. 8:**
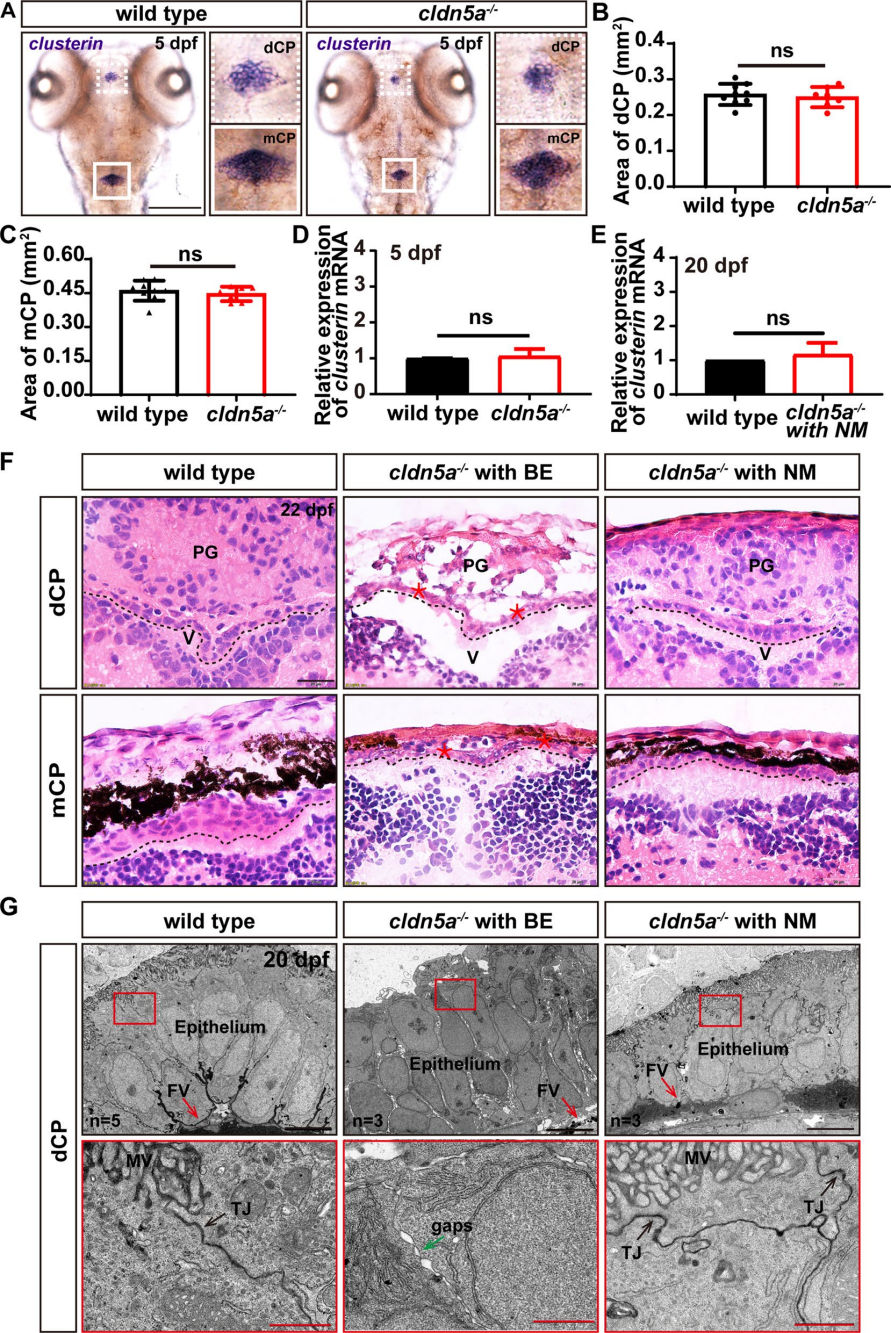
Development and morphology of CPs in *cldn5a*^*-/-*^. **A** WISH of *clusterin* reflects normal morphology of CPs in 5 dpf larvae of *cldn5a*^*-/-*^. White dash line squares and solid squares indicate the regions of dCPs and mCPs which are shown in high magnification in the right panels respectively. Scale bars: 200 μm. **B**, **C** The area of dCPs and mCPs were quantified and no difference between wild types and *cldn5a*^*-/-*^ embryos was detected. **D**, **E** The expression levels of *clusterin* mRNA in 5 dpf embryos and 20 dpf larvae was analyzed by RT-qPCR and no discrepancy was detected between wild type and *cldn5a*. Each experiment was repeated more than three times. Data are shown as mean ± SEM. ns meant no significance. **F** Histological analysis by HE staining was performed to show the morphology dCPs and mCPs in 22 dpf larvae. In the brains of *cldn5a*^*-/-*^ with NM, the morphology of CP epithelium is as normal as in wild types. In *cldn5a*^*-/-*^ with BE, the structure of the pineal gland (PG) is disorganized with irregular cell arrangement and severe cell necrosis. The epithelial cells of dCPs and mCPs were necrotic and the plexus epithelial layer is disordered. Meanwhile, the brain ventricles (V) of *cldn5a*^*-/-*^ with BE were enlarged dramatically. Black dot lines figure out the linear CP epithelium. The necrotic CP epithelial cells are indicated by red asterisks. n = 3 fishes analyzed per group. Scale bars: 20 μm. **G** Ultrastructural analysis of dCP epithelium of wild types and *cldn5a*^*-/-*^ with or without BE at 20 dpf. The morphology of cerebral vascular ECs and the integrity of TJs in *cldn5a* with NM are normal as in wild types. The morphology of dCP epithelium and paracellular TJs (black arrows) in *cldn5a* with NM was not altered compared to that in wild types, whereas the microvillus of the dCP epithelial cells disappeared in *cldn5a*^*-/-*^ with BE. Moreover, there occured paracellular gaps between dCP epithelial cells (green arrow). The regions labeled by red rectangles show epithelial TJs which are presented in the lower panel with high magnification. MV, microvilli; FV, fenestrated vessel. n > 3 fishes analyzed per group. ns means no significance. Scale bars: 5 μm in black and 1 μm in red

In parallel to the investigation of cerebral vessels, the morphology and development of CP in *cldn5a*^*-/-*^ were analyzed. The WISH of *clusterin* on 5 dpf embryos indicated that the morphology and the sizes of both dCP and mCP were not altered in *cldn5a*^*-/-*^ (Fig. 8A–C). Meanwhile, as shown in Fig. 8D and E, there were no significant differences in the expression level of *clusterin* between the *cldn5a*^*−/*−-/-^ and the wild type at both 5 dpf and 20 dpf stages. We next analyzed the morphology of CP epithelium by histological sections through the brains of 22 dpf larvae. As shown in Fig. 8F, in the brain of *cldn5a*^*-/-*^ with NM, the morphology of CP epithelium lining above the brain ventricle was intact, similar to that in wild types. Besides, the ultrastructural analysis on 20 dpf-old larval dCP showed an intact CP epithelium and epithelial TJs in both wild types and *cldn5a*^*-/-*^ with NM brains (black arrows in Fig. 8G). In contrast, in *cldn5a*^*-/-*^ with BE, the ventricular cavity under the dCP was enlarged obviously (Fig. 8F). Moreover, the pineal gland was disrupted with severe cell necrosis, and the structure of CP epithelium was disordered (red asterisks in Fig. 8F). Meanwhile, the ultrastructural analysis of the CP epithelium in *cldn5a*^*-/-*^ with BE revealed that the microvillus of CP epithelial cells disappeared, and there showed obvious paracellular gaps between adjacent epithelial cells (Fig. 8G). These could be a direct consequence of cerebral inflammation or neuronal apoptosis caused by the leakage of brain barriers.

Since the notch and shh pathway had been reported to regulate the development of zebrafish CP [38, 39], we next analyzed the expression levels of several genes related to notch and shh pathways in *cldn5a*^*-/-*^ at different developmental stages. As shown in Additional file 1: Fig. S11, there was no obvious difference in the expressions of these genes between wild types and *cldn5a*^*-/-*^ with NM brains either at the embryonic stage or larval stage (Additional file 1: Fig. S11A, B and D, E). However, downregulation of several notch or shh pathway genes was detected including *gli2a, notch1b*, *-2*, *-3*, and *jagged2a*, *-2b* in *cldn5a*^*-/-*^ with BE, probably due to the severe neuronal apoptosis in the larvae (Additional file 1: Fig. S11C, F).

### Upregulation of ZO-1 in the choroid plexus of larval *cldn5a*^*−/−*^

**Fig. 9:**
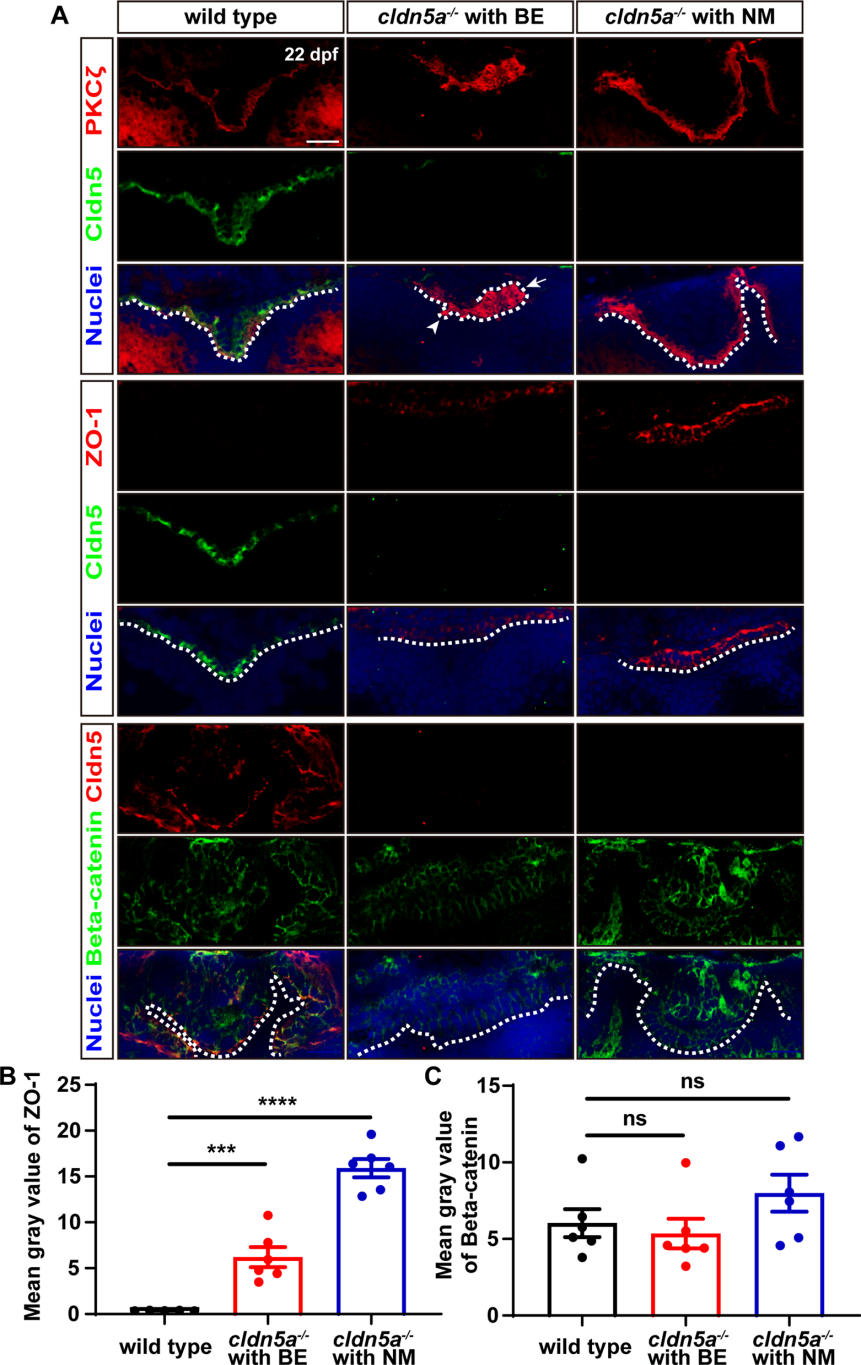
Maintenance of CP epithelial polarity and junctions in *cldn5a*^*-/-*^ larvae. **A** Apical polarity marker of PKCζ and epithelial junction proteins were stained to investigate the integrity of dCP epithelium. In the larval brains of wild types and *cldn5a*^*-/-*^ with NM, PKCζ colocalizes with Cldn5a and is linearly expressed in the apical side of dCP epithelium. In *cldn5a*^*-/-*^ with BE, PKCζ localizes non-apically and misexpresses on the protruded CP into the ventricle (white arrowhead) and the misfolded plexus epithelial layers (white arrow). Furthermore, compared to that in wild types, *cldn5a*^*-/-*^ larvae show a dramatic upregulation of ZO-1. There was no discrepancy in the expression of Beta-catenin between wild types and *cldn5a*^*-/-*^ mutated larvae. White dot lines figure out the linear dCP epithelium. n > 3 fishes analyzed per group. Scale bars: 20 μm. **B**, **C** The quantitative analysis of ZO-1 and Beta-catenin by measuring the mean gray values in CP epithelial cells with ImageJ software. Data are shown as mean ± SEM. ***P < 0.005, ****P < 0.001

Since the above results indicated that the development of zebrafish CP was not affected in the absence of Cldn5a, we asked whether there exist molecular compensation effects during the formation of CP in *cldn5a*^*-/-*^. To this end, other junctional proteins and the cell polarity factors of CP epithelium were investigated firstly since the polarity is the significant character of CP epithelium [40]. As shown in Fig. 9A, in the larval CP of *cldn5a*^*-/-*^ with NM brain, the epithelial cell polarity marker of PKCζ localized in the apical CP epithelium together with Cldn5a, as its expression and localization in wild type CPs. In contrast, misexpressed and non-apically localized PKCζ was found in the CPs of *cldn5a*^*-/-*^ with BE, with the occurence of abnormally misfolded plexus epithelial layers and protruded CP cells into the ventricle (Fig. 9A). Next, the expression of TJ protein of ZO-1 and adherent junction protein of Beta-catenin in zebrafish CP were analyzed by IF staining. Surprisingly, we found that ZO-1 was dramatically upregulated in both *cldn5a*^*-/-*^ brains with BE and NM when compared with that in wild types (Fig. 9A, B). However, as to the expression of Beta-catenin in CPs of 22 dpf larvae, there was no difference of its expression between the CPs of wild types and *cldn5a*^*-/-*^ with or without BE (Fig. 9A, C). Together, these data suggest that the TJ protein of ZO-1 might be compensatorily increased in the CPs of *cldn5a*^*-/-*^ .

**Fig. 10:**
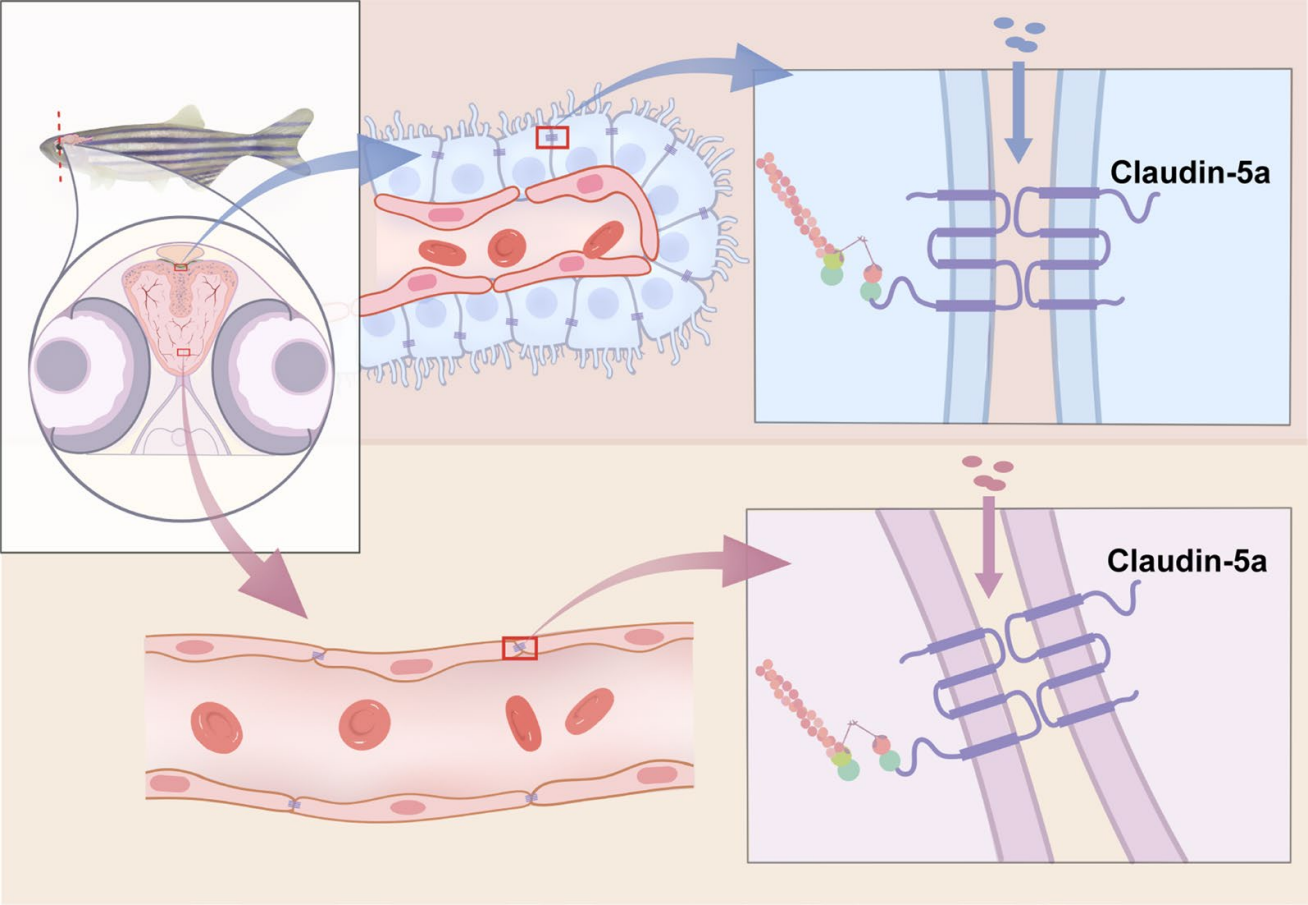
Cldn5a is essential for the development of both zebrafish BBB and BCSFB. Zebrafish Cldn5a expresses in both cerebrovascular endothelial cells and CP epithelial cells and is required for the functional maintenance of the barrier function of BBB and BCSFB

In general, our findings revealed the essential roles of the TJ protein Cldn5a on the functional maintenance of both zebrafish BBB and BCSFB (Fig. 10). Meanwhile, although zebrafish Cldn5b is expressed in cerebral ECs as well, it showed little effect on the development of zebrafish vasculatures and functional maintenance of BBB.

## Discussion

In mammals, more than 23 CLDNs are expressed in diverse tissues and play distinct physiological functions [14]. Naturally, barrier forming CLDNs in BBB endothelium or BCSFB epithelium limit the transport of detrimental molecules between blood and brain or between blood and CSF [41]. Among them, CLDN1 and CLDN3 are strongly expressed in CP epithelium [29, 42–45], and CLDN1 is expressed not only in cerebral parenchymal microvessels but also in meningeal blood vessels [44, 45]. However, whether CLDN3 is expressed in cerebrovascular endothelium is controversial [16, 29, 45, 46]. Recently, Castro Dias et al. reported that CLDN3 is not expressed in BBB endothelium as evidenced by generating *Cldn3*^*-/-*^ mice and single-cell RNA-sequencing analysis [29]. They conclude that previous studies supporting the expression of CLDN3 in BBB ECs were due to the cross-reactivity of widely used anti-CLDN3 antibodies [29]. CLDN5 is conservatively expressed in the CNS vascular endothelium of multiple species [9, 19, 45, 47, 48]. Nevertheless, unlike mammalian CLDN5, zebrafish Cldn5a is also expressed in CP epithelium [9]. Here, we detected CLDN5 only expression in human and mouse cerebral vessels but not in CP epithelium. Meanwhile, we observed the distribution of CLDN1 and CLDN3 in CP epithelium. Probably due to the cross-reactivity of anti-CLDN3 antibodies [19, 29], we also found CLDN3 in human and mouse cerebrovascular ECs. To further verify the zebrafish orthologous gene of mammalian *CLDN5*s, we analyzed several representative CLDN proteins from human, mouse, and zebrafish by phylogenetic trees. As expected, we identified zebrafish *cldn5a* as the ortholog of mammalian *CLDN5*s.

In zebrafish there exist two paralogous *cldn5*s, *cldn5a,* and *cldn5b*, with distinct expression patterns [21]. During the early embryogenesis, both *cldn5a* mRNA and *cldn5b* mRNA are expressed in the entire vascular system while *cldn5a* transgenic lines show that Cldn5a is also distributed in CP [9, 22, 24, 25]. Consistent with these previous findings, our results revealed that *cldn5b* is ubiquitously expressed in the heart, liver, and brain of adult zebrafish, while *cldn5a* shows a surprisingly higher expression in the adult brain than in other tissues. Due to the limitation of specific antibodies which could distinguish Cldn5a and Cldn5b, there were no reports that could accurately characterize the protein expression pattern of Cldn5a and Cldn5b in zebrafish. Also, whether there is a genetic complementary effect between these two genes was unclear. To solve these questions, we generated *cldn5a* and *cldn5b* zebrafish mutants respectively [25]. For the first time, we identified that there is no genetic complementary effect between *cldn5a* and *cldn5b* during the early and late developmental stages of zebrafish. Utilizing *Tg(kdrl:EGFP)* line, we also clarified for the first time the protein expression patterns of Cldn5a and Cldn5b that Cldn5a expresses in both cerebral vessels and CP epithelial cells, and Cldn5b distributes only in cerebral ECs.

It has been reported that CLDN5 is required for establishing tissue barriers in vivo and in vitro. The epithelial barrier property of Caco-2 cells, a human colonic cell line, is increased by stably expressing exogenous CLDN5 [49]. MDCK-II transfectants expressing mammalian CLDN5 could strengthen the paracellular barrier [50]. Moreover, the loss of CLDN5 results in the leakage of the BBB in vivo and in vitro [19, 51]. Here, although the transfections of zebrafish-specific *cldn5a* into in vitro cells were missing, we verified that mammalian CLDN5 is required for establishing both epithelial and endothelial barriers in multiple cell models, consistent with its canonical sealing functions reported (Additional file 1: Fig. S9F)*. CLDN5*-knocked out bEnd.3 cells and HBMEC cells showed disruptions of endothelial barrier while expression of exogenous *CLDN5* in MDCK-I cell strengthens epithelial barrier, suggesting that CLDN5 is essential for forming and maintaining the epithelial and endothelial barrier. Cldn5a, as the ortholog of mammalian CLDN5, has been reported to play an important role in establishing and maintaining multiple brain barriers during early embryonic development. Zhang et al. reported that Cldn5a is essential for brain ventricle expansion by forming a neuroepithelial barrier [23]. A study by Ahn et al. indicated that Cldn5a was required for forming and maintaining the integrity of the blood-neural barrier. *cldn5a* morphants showed a size-selective loosening of zebrafish BBB and the decrease of the glucose transporter 1 mRNA (*glut1*) expression in the cerebral microvessels [24]. Furthermore, suppressing *cldn5a* with morpholinos caused a temporary breakdown of BCSFB and the arachnoid barrier [24]. However, the knockdown effect of morpholinos is time-dependent, and *cldn5a* morphants showed increased levels of apoptosis [24]. Therefore, it is difficult to rule out whether the increased level of apoptosis is derived from the unexpected adverse effects of morpholinos, which could indirectly break down the brain barrier. Moreover, it might be inappropriate to assess the integrity of brain barriers with morphants at 7 dpf. Although the maturation time of zebrafish BCSFB is 4 days [8], the arachnoid barrier matured after 9 days [7]. Umans et al. determined that barriergenesis occurs simultaneously with angiogenesis [52]. The time of BBB maturation in zebrafish is debatable, varying from 3 to 10 dpf [7, 11, 53, 54]. Here, *cldn5a* and *cldn5b* mutants were introduced to better clarify the specific roles of *cldn5a* and *cldn5b* in zebrafish brain barriers. We observed that *cldn5a*^*-/-*^ showed a fatal BE phenotype at 20-30 dpf with severe whole-brain oedema, ventricular dilatation, and cerebral hernia, and *cldn5a*^*-/-*^ showed the breakdown of zebrafish BBB and BCSFB, in all fore-, mid- and hindbrains (Figs. 4, 5 and 6). In contrast, we found that *cldn5b*^*-/-*^ showed normal morphology and intact BBB. Therefore, Cldn5b might play an unessential role in zebrafish development since it is only expressed in a few cerebral vessels and its absence might be compensated by Cldn5a or other TJ proteins. This may also explain why *cldn5b* is obsoleted in mammals. As mentioned above, Cldn5a expresses in most cerebrovascular endothelium during the late zebrafish developmental stages. In other words, *cldn5a* but not *cldn5b* is essential for the establishment and maintenance of zebrafish brain barriers.

Moreover, in our study, we found that except for partial *cldn5a*^*-/-*^ which died at 24-30 hpf due to vasculogenesis defects as we reported [25], most of the other *cldn5a*^*-/-*^ survived from the embryos till the larval stages around 25 dpf, indicating a non-lethal effect of leak BBB and BCSFB. This may remind that Cldn5a and its contributed BBB and BCSFB are getting more and more important in maintaining cerebral barrier functions as the zebrafish develops. To some extent, this could also explain the inconsistent findings between *cldn5a* morphant and *cldn5a*-knocked out zebrafish where 10 kDa-rhodamine dextran is still retained within cerebral microvessels in knockdown zebrafish at 7 dpf, but leaking in *cldn5a* knockouts during the late developmental stages [24].

BE, cerebral inflammation, and apoptosis are usually concomitant and interactive pathophysiological processes implicated in many CNS diseases [32–34]. Breakdown of brain barriers causes a large amount of blood detrimental molecules extravasating from cerebral vessels into the brain parenchyma or getting rid of the restriction of CP epithelium and intruding into the ventricle. These stimulations lead to the activation of microglia cells and further cause cerebral inflammation. Both brain oedema and CNS inflammation could result in cerebral apoptosis. Typical cases are neurological dysfunctions caused by the breakdown of BBB and the formation of edema in acute and chronic cerebral ischemia [55]. On the other hand, in some CNS diseases, neuroinflammation and cell death could in turn lead to further destructions of the brain barriers, such as in multiple sclerosis (MS) [56]. These might explain the cerebral inflammation and neuronal apoptosis in *cldn5a*^*-/-*^ with BE. Pro-inflammatory cytokines of *il-1b* and *il-8*, especially *il-1b*, were up-regulated dramatically in the brains of *cldn5a* mutants with BE. The increased expression of *il-1b* and *il-8* might be due to the activation of microglial cells [35, 36]. Meanwhile, we observed that the distribution of microglia cells was altered in *cldn5a* mutants with BE compared with that in wild types. The number of microglia decreased significantly in the CPs of mutants with BE. We considered that active microglia cells in CPs of *cldn5a*^*-/-*^ with BE might be recruited to the lesion sites of other brain regions due to severe edema and extensive neuronal apoptosis in the whole cerebral parenchyma. Except for neuroinflammation, we found that a large amount of cerebral apoptosis have already taken place in the whole brain of *cldn5a*^*-/-*^ with BE at around 20 dpf (Additional file 1: Fig. S7A-E). These serious cerebral inflammation and apoptosis might in turn deteriorate and disrupt the brain barriers further [56]. In this study, it was found that the tightness of BBB and BCSFB has already been disrupted in the brains of *cldn5a*^*-/-*^ with NM at 20 dpf stage. Moreover, since *cldn5a*^*-/-*^ with BE showed destructive brain histology and severe neuronal apoptosis (Figs. 3, 7D and Additional file 1: Fig. S7), we infer that these mutants have defective cerebral barriers without doubt. Therefore, we did not use the *cldn5a*^*-/-*^ with BE to further assess their brain barrier functions. Besides, the severely disorganized brain histology in the *cldn5a*^*-/-*^ with BE also technically limited the functional analyses of their cerebral barriers (Figs. 3 and 7D).

In CLDN5-deficient mouse embryos, the development and the morphology of cerebral vessels were normal [19]. In our previous study, we found that the vasculogenesis of *cldn5b*^*-/-*^ was not altered, while the lumenization of the dorsal aorta in *cldn5a*^*-/-*^ was defective [25]. Here, we systematically analyzed the development of CP and cerebrovasculatures in *cldn5a*^*-/-*^ during the early and late development and found that both of them were normal. In *cldn5a*^*-/-*^, the cerebral vasculogenesis was intact and the expression of Wnt pathway factors was not affected. Moreover, the ultrastructure of cerebral capillaries and the TJ integrity at the endothelial cell–cell contact regions were normal too. In other words, loss of *cldn5a* or *cldn5b* would not affect the development of cerebral vessels. As shown in Additional file 1: Fig. S10, the growth properties of cerebral vascular ECs were not affected by the loss of CLDN5. Meanwhile, compared with those in wild types, except for an increased expression of ZO-1 in *cldn5a*^*-/-*^ CP epithelium, the expression level of the notch and shh pathways regulating CPs development, the histological morphology and ultrastructure of CPs, and the polarity of CP epithelium were all not altered. In the *CLDN5*-deficient mice brain, the existence of the CLDN12-based TJs would keep the structural integrity of BBB endothelium [19]. In the *cldn5a*^*-/-*^ zebrafish CP, the integrity of TJ structure might be partially maintained by the compensatory increase of ZO-1 or other undetected Cldns during the early embryonic stage.

The development of CP and cerebral vessels was not affected by the loss of Cldn5a during embryonic stages. In the *cldn5a*^*-/-*^ larvae, it is hard to determine exactly the disruption of which barrier or both of them caused the lethal of the mutants since Cldn5a expresses in both BCSFB-dependent CP epithelial cells and BBB-dependent cerebrovascular ECs. To answer this question, generating conditionally knocked-out *cldn5a* zebrafish in cerebral vessels or CP epithelium respectively, or rescuing *cldn5a*^*-/-*^ by tissue-specific expression of *cldn5a* in cerebral vascular endothelium or CP epithelium might be necessary.

## Conclusion

We verified an inoperative role of zebrafish Cldn5b in the development and functional maintenance of BBB, and the essential roles of Cldn5a in the functional formation of both zebrafish BBB and BCSFB (Fig. 10).

## Supplementary Information

**Additional file 1: Figure S1.** Expression of CLDNs in human and mice brain vessels and choroid plexuses. **Figure S2.** Phylogenetic tree of several related Claudins from human, mouse and zebrafish. **Figure S3.** Expression levels and patterns of* cldn5a* and *cldn5b* in zebrafish. **Figure S4.** Expression patterns of Cldn5a and Cldn5b in zebrafish brain. **Figure S5.** Dilatation of midbrain ventricles in *cldn5a*^*-/-*^. **Figure S6.** Cerebral inflammation in the brains of *cldn5a*^*-/-*^ with BE. **Figure S7.** Cell apoptosis in the brains of 20 dpf larvae. **Figure S8.** Detection of endogenous biotin in zebrafish brain. **Figure S9.** CLDN5 is important for endothelial or epithelial barriers*.* **Figure S10.** Loss of CLDN5 in endothelial cells shows no effects on cell proliferation and apoptosis. **Figure S11.** The expression levels of genes for notch and shh pathways in *cldn5a*^*-/-*^ are not affected. **Table S1.** Primers used for PCR, RT-PCR, and RT-qPCR. **Table S2.** Primary and secondary antibodies used for IHC and IF staining.

**Additional file 2: Movie S1.** Swimming behavior movie of *cldn5a*^*-/-*^ larvae at 20 dpf.

**Additional file 3: Movie S2.** Swimming behavior movie of *cldn5a*^*-/-*^ at 42 dpf.

## Data Availability

The datasets analyzed during the current study are available from the corresponding author on reasonable request.

## Abbreviations

BBB: Blood-brain barrier
BCSFB: Blood-cerebrospinal fluid barrier
BE: Brain edema
Cldn: Claudin
CNS: Central nervous system
CP: Choroid plexus
dCP: Diencephalic choroid plexus
mCP: Myelencephalic choroid plexus
NM: Normal morphology
TJ: Tight junction
