# Supplementary material for: Claudin-5a is essential for the functional formation of both zebrafish blood-brain barrier and blood-cerebrospinal fluid barrier
Source: Fluids Barriers CNS. 2022 Jun 3;19:40. doi: 10.1186/s12987-022-00337-9 (PMC9164509; doi:10.1186/s12987-022-00337-9)
Supplement: Supplementary file 1 — Additional file 1: Figure S1. Expression of CLDNs in human and mice brain vessels and choroid plexuses. Figure S2. Phylogenetic tree of several related Claudins from human, mouse and zebrafish. Figure S3. Expression levels and patterns of cldn5a and cldn5b in zebrafish. Figure S4. Expression patterns of Cldn5a and Cldn5b in zebrafish brain. Figure S5. Dilatation of midbrain ventricles in cldn5a-/-. Figure S6. Cerebral inflammation in the brains of cldn5a-/- with BE. Figure S7. Cell apoptosis in the brains of 20 dpf larvae. Figure S8. Detection of endogenous biotin in zebrafish brain. Figure S9. CLDN5 is important for endothelial or epithelial barriers. Figure S10. Loss of CLDN5 in endothelial cells shows no effects on cell proliferation and apoptosis. Figure S11. The expression levels of genes for notch and shh pathways in cldn5a-/- are not affected. Table S1. Primers used for PCR, RT-PCR, and RT-qPCR. Table S2. Primary and secondary antibodies used for IHC and IF staining. [file 12987_2022_337_MOESM1_ESM.docx]

**Additional Information**

**Additional Figures & Legends**

**
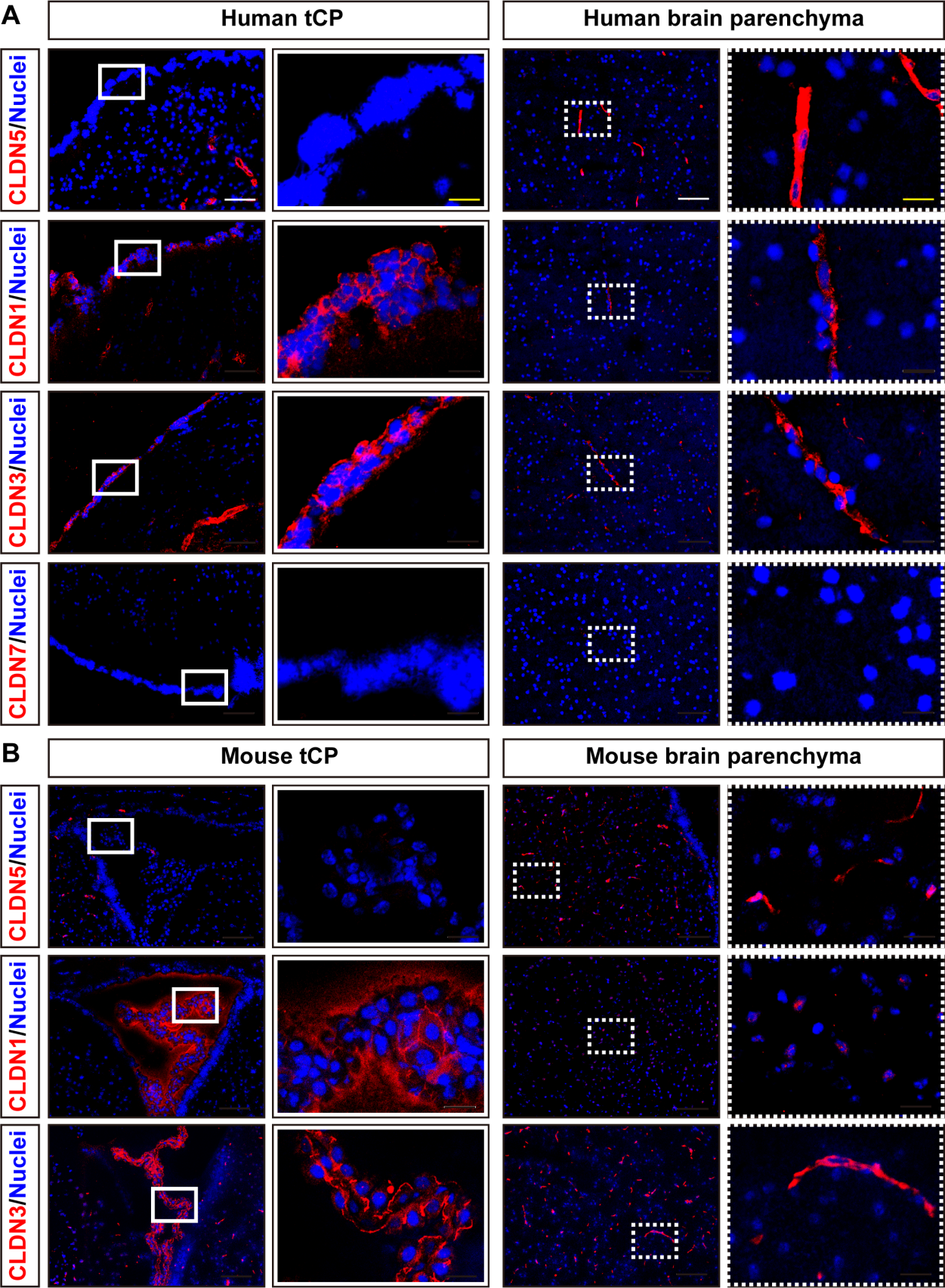
**

**Figure S1. Expression of CLDNs in human and mice brain** **vessels and choroid plexuses.** (A) CLDN5 is strongly expressed in human cerebrovascular ECs but not in human CP. CLDN1 and CLDN3 are mainly expressed in human CP epithelium. CLDN7 shows no expression in human CP or human brain parenchyma. (B) CLDN5 is strongly expressed in mice cerebrovascular ECs but not in mice CP. CLDN1 and CLDN3 are mainly expressed in mice CP epithelium. To be noticed, CLDN1 and CLDN3 signals are also detected in human and mice cerebral vessels probably due to the cross-reactivity of antibodies. White rectangles or dashed rectangles indicate the enlarged regions of the human CP and mouse tCP (telencephalic choroid plexus), or human and mouse cerebral vessels respectively which are shown in the right panels with high magnification. n = 3 samples analyzed per group. Scale bars, 100 μm in white or 20 μm in yellow.


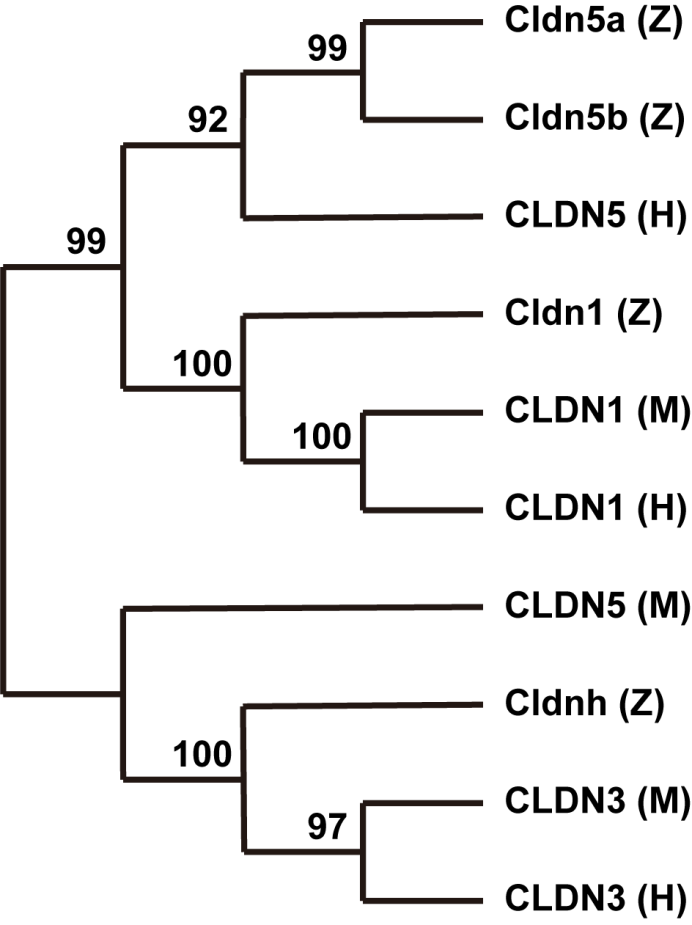


**Figure S2. Phylogenetic tree of several related Claudins from human, mouse and zebrafish.** A rooted phylogenetic tree was created to show the evolutionary relationships between the Cldns from human, mouse, and zebrafish. Cldn sequences used include human CLDN1 (NP_066924.1), CLDN3 (NP_001297.1), CLDN5 (NP_001349995.1), mouse CLDN1 (NP_057883.1), CLDN3 (NP_034032.1), CLDN5 (NP_038833.2), and zebrafish Cldn1 (NP_571845.1), Cldnh (AAL01841.1), Cldn5a (NP_998439.1) and Cldn5b (NP_001006044.2). Bootstrap values on the blanches represent the phylogenetical distances calculated according to the ClustalW slow/accurate method. H, human; M, mouse; Z, zebrafish.

**
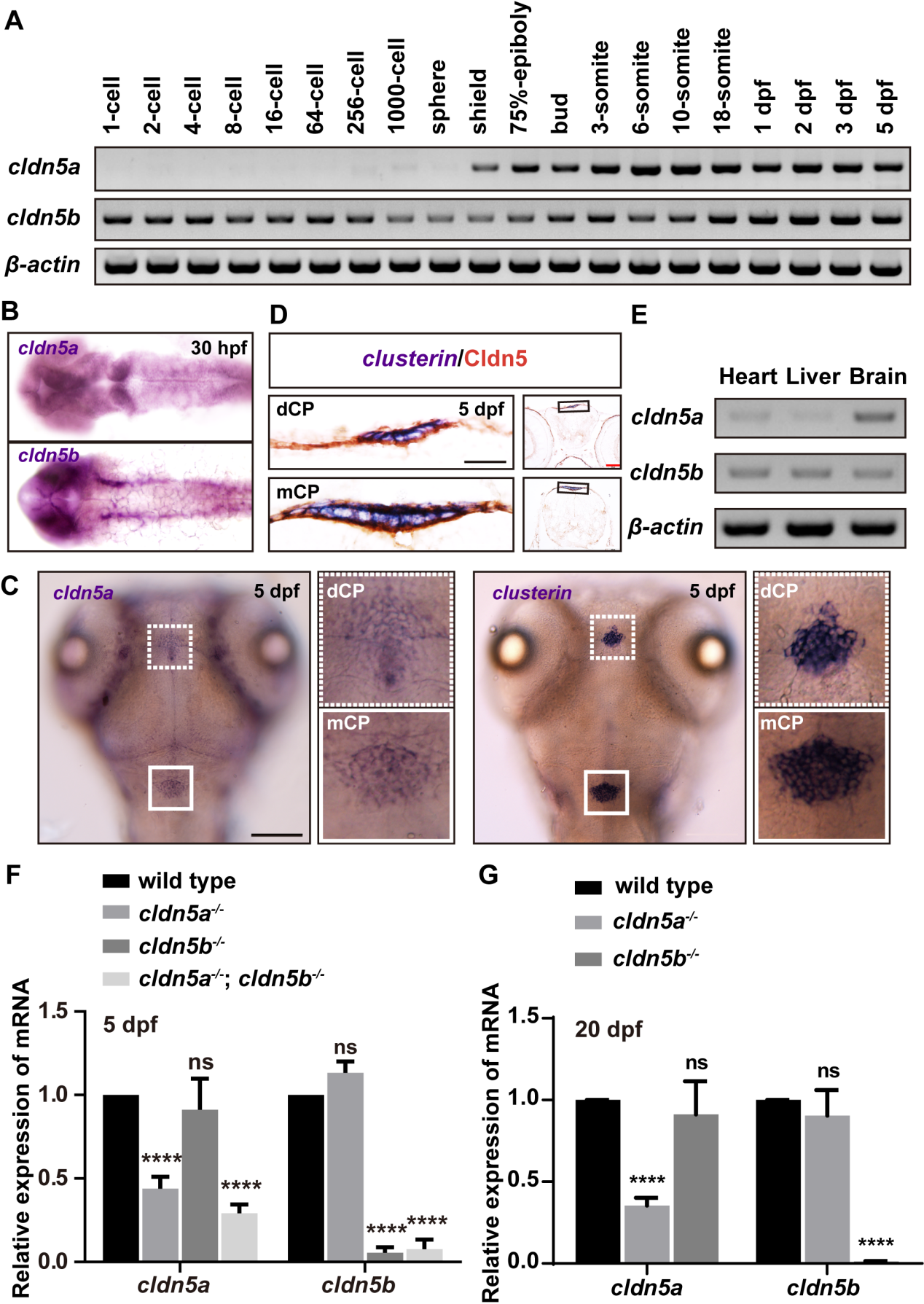
**

**Figure S3. Expression levels and patterns of *cldn5a* and *cldn5b* in zebrafish.** (A) Expression levels of total *cldn5a* and *cldn5b* mRNA from 1-cell stage to 5 dpf embryos. The level of *β-actin* was used as a control. (B) WISH of *cldn5a* and *cldn5b* in the brains of 30 hpf embryos. (C) WISH of *cldn5a* and *clusterin* as a marker of CPs in 5 dpf embryos. *clusterin* and *cldn5a* are both expressed in dCP (white dashed square) and mCP (white solid square). Enlarged images of the dCP and mCP regions are shown in the right panels. Scale bars, 100 μm. (D) After WISH of *clusterin*, IHC staining of Cldn5 was performed on the brain sections to display its expression on CP epithelial cells. Panoramic images were shown in right side. Black rectangles in the right panels show the regions of dCP and mCP, which are presented in the left panels in high magnification. Scale bars: 20 μm in black and 50 μm in red. (E) Expression levels of total *cldn5a* and *cldn5b* mRNA from heart, liver, and brain of adult zebrafish were analyzed by RT-PCR. *β-actin* was loaded as a control. (F, G) Expression levels of *cldn5a* and *cldn5b* in the brains of wild-types, *cldn5a^-/-^*, *cldn5b^-/-^* and *cldn5a^-/-^*;*cldn5b^-/-^* at developmental stages of 5 dpf and 20 dpf respectively. Data are presented as the mean ± SEM. Experiments were repeated more than three times. **** P<0.001; ns means no significance.


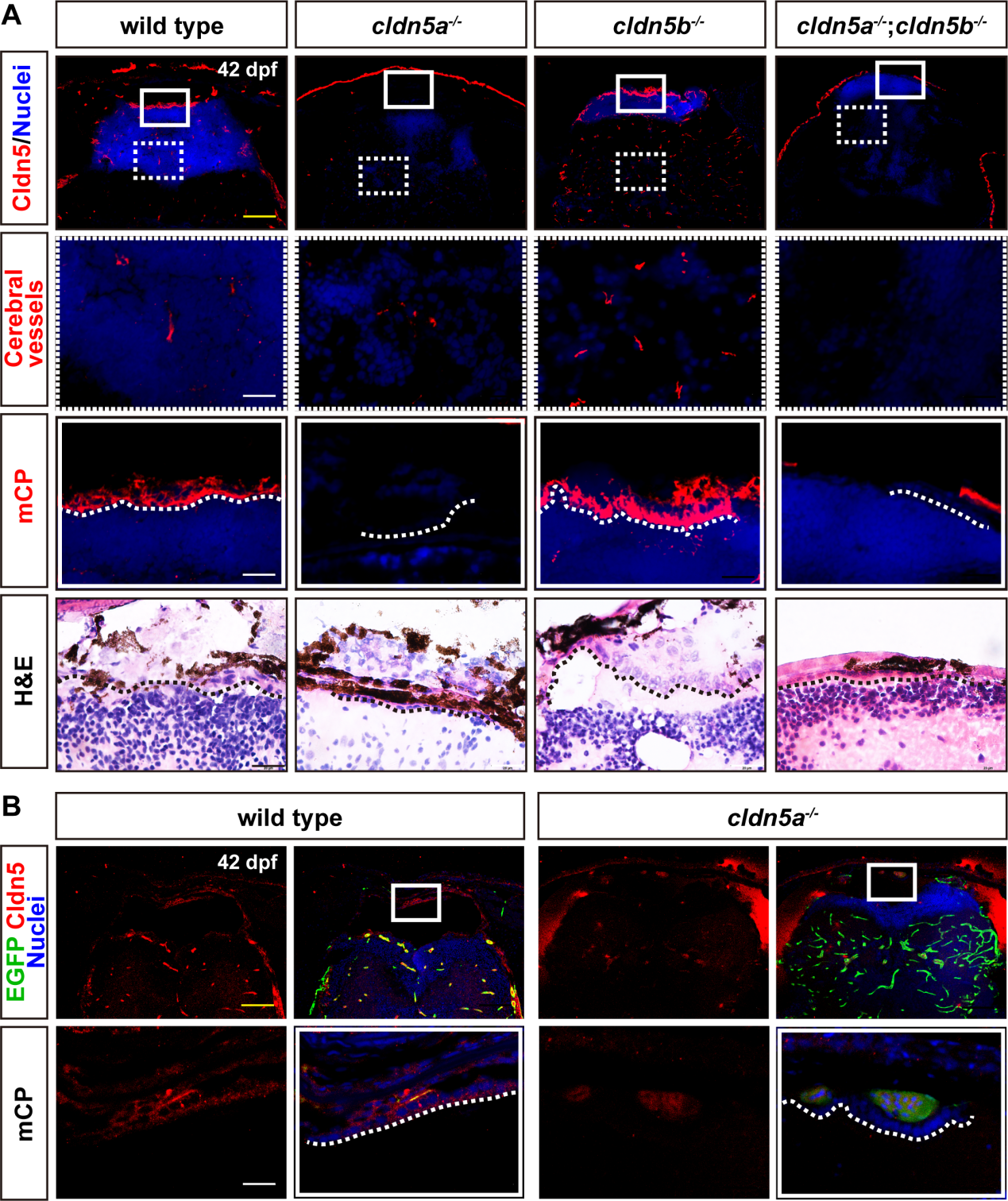


**Figure S4. Expression patterns of Cldn5a and Cldn5b in zebrafish brain.** The brain sections of 42 dpf-old zebrafish were stained by anti-pan Cldn5 antibody. (A) In wild types, Cldn5s are not only expressed in cerebrovascular ECs but also in the mCP epithelium. In *cldn5a^-/-^*, Cldn5b expresses only in few cerebrovascular ECs but not in mCP. In *cldn5b^-/-^*, Cldn5a is strongly expressed in both cerebrovascular ECs and mCP. In *cldn5a^-/-^*;*cldn5b^-/-^*, no specific signals could be detected in brain. Serial sections were stained with HE to show the histology of mCP. (B) *Tg(kdrl:EGFP)* zebrafish was introduced to help to determine the endothelial Cldn5 expression. In *cldn5a^-/-^*, Cldn5b is only expressed within few cerebral vessels but not in mCP epithelium. White rectangles or dashed rectangles indicate the enlarged regions of mCP, or cerebral vessels respectively which are shown in the lower panels with high magnification. White dotted lines or black dotted lines show the continuous mCP epithelium. n = 3 fishes analyzed per group. Scale bars, 100 μm in yellow and 20 μm in black or white.


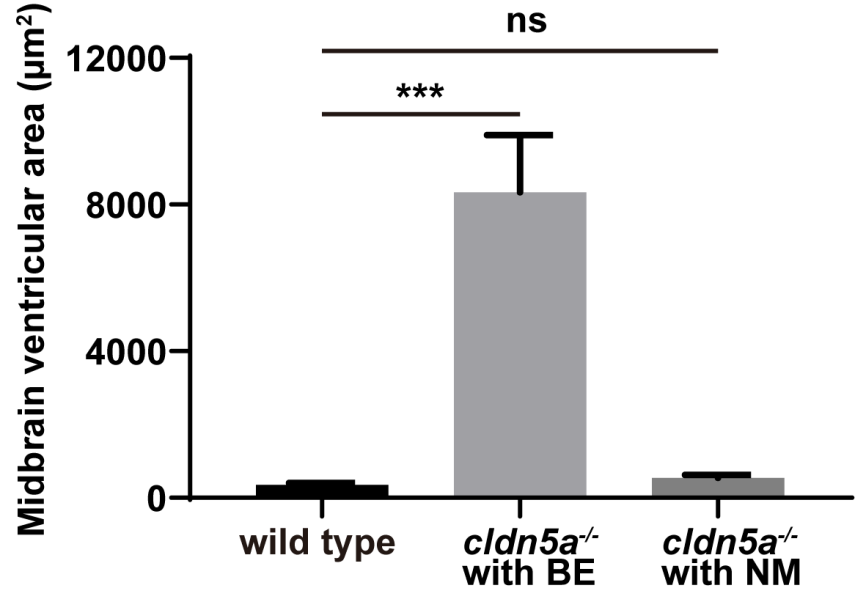


**Figure S5. Dilatation of midbrain ventricles in *cldn5a^-/-^*.** After HE staining on serial brain sections, the area of midbrain ventricle was measured and quantitatively analyzed by ImageJ software.

**
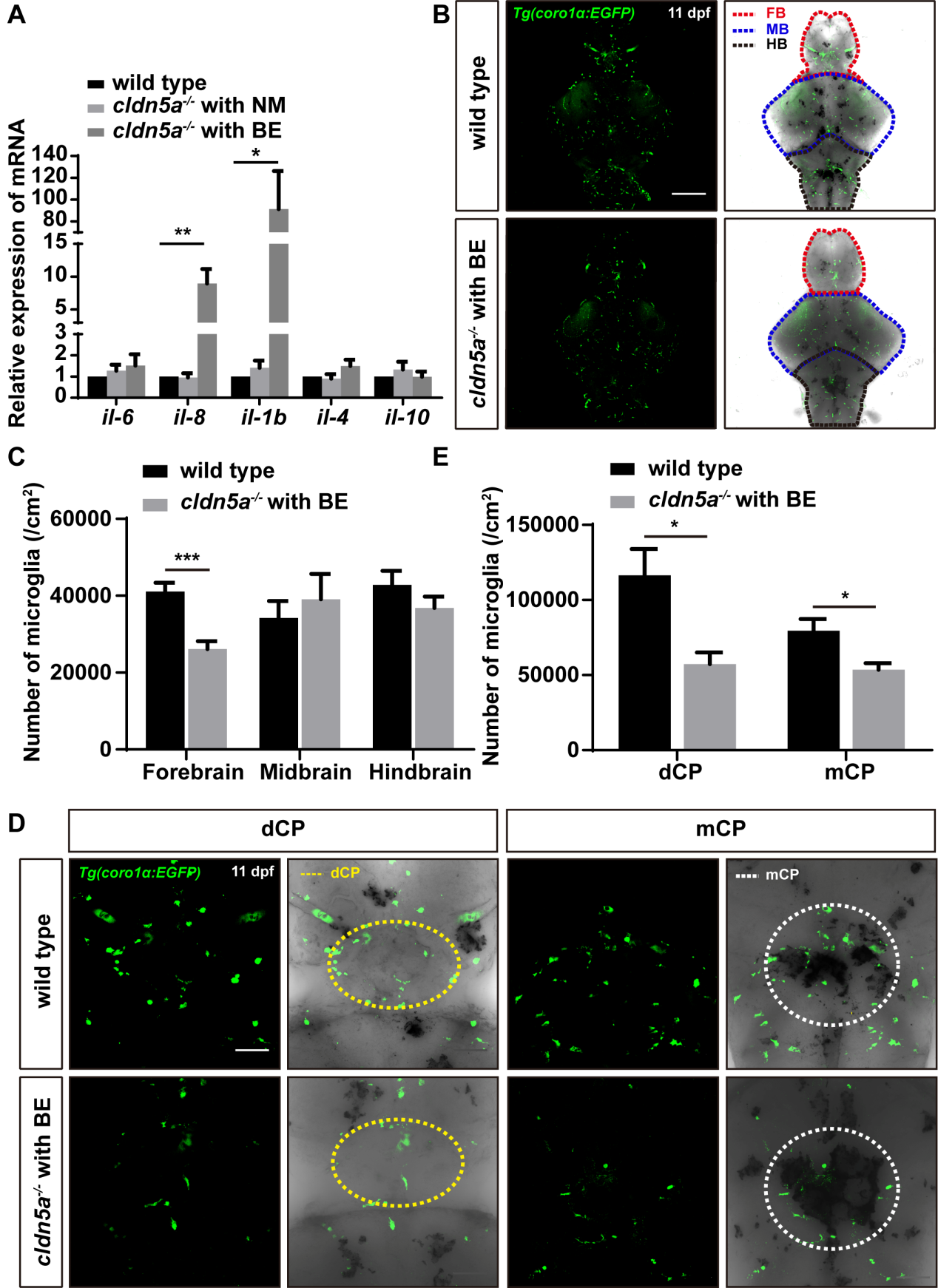
**

**Figure S6. Cerebral inflammation in the brains of *cldn5a^-/-^* with BE.** (A) Gene expression levels of pro-inflammatory cytokines (*il-6*, *il-8*, *il-1β*) and anti-inflammatory cytokines (*il-4* and *il-10*) were detected by RT-qPCR. (B, C) Transgenic line of *Tg(coro1α:eGFP)* in which macrophages are labeled by GFP was applied to evaluate the cerebral inflammation. The number of microglia in different brain regions of wild-types and *cldn5a^-/-^* with BE was quantified. Red dot line circles label the forebrain (FB). Blue dot line circles label the midbrain (MB). Black dot line circles label the hindbrain (HB). Scale bars: 200 μm. (D, E) The numbers of microglia cells in the dCP and mCP of wild-types and *cldn5a^-/-^* with BE were quantified. Yellow dot circles represent dCP. White dot circles represent mCP. Scale bars, 50 μm. n = 3 fishes analyzed per group. Data are shown as mean ± SEM. * P< 0.05, ** P<0.01, *** P<0.005.

**
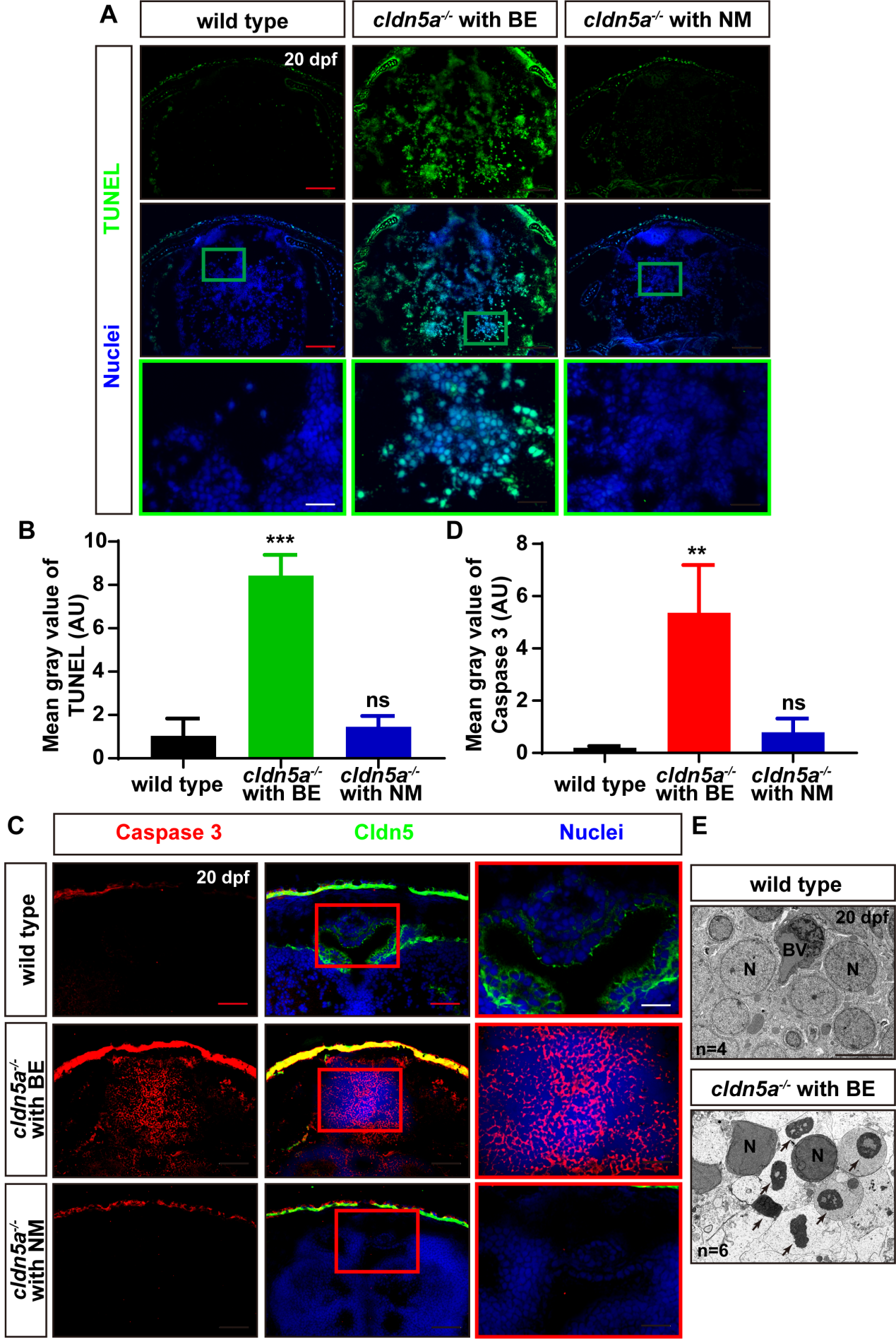
**

**Figure S7. Cell apoptosis in the brains of 20 dpf larvae.** (A, B) TUNEL assay was performed on brain sections to detect apoptosis in 20 dpf larval brains of wild type siblings, *cldn5a^-/-^* with BE, and *cldn5a^-/-^* with NM respectively. Green rectangles indicate the enlarged brain regions which are shown in the lower panels with high magnification. Scale bars, 100 μm in yellow or 20 μm in white. (C, D) Caspase 3 was detected to reflect apoptosis of brains of 20 dpf-old wild type siblings, *cldn5a^-/-^* with BE, and *cldn5a^-/-^* with NM respectively. Red rectangles indicate the enlarged brain regions which are shown in the right panels with high magnification. Scale bars, 50 μm in red or 20 μm in white. (E) Ultrastructural analysis on the sections of zebrafish brain parenchyma of wild-types and *cldn5a^-/-^* with BE at 20 dpf. BV, blood vessel; N, neuron. Black arrows indicate the nucleus of apoptotic neurons. Scale bars, 5 μm. n > 4 fishes analyzed per group. Data are represented as mean ± SEM. ** P<0.01, *** P<0.005. ns means no significance.


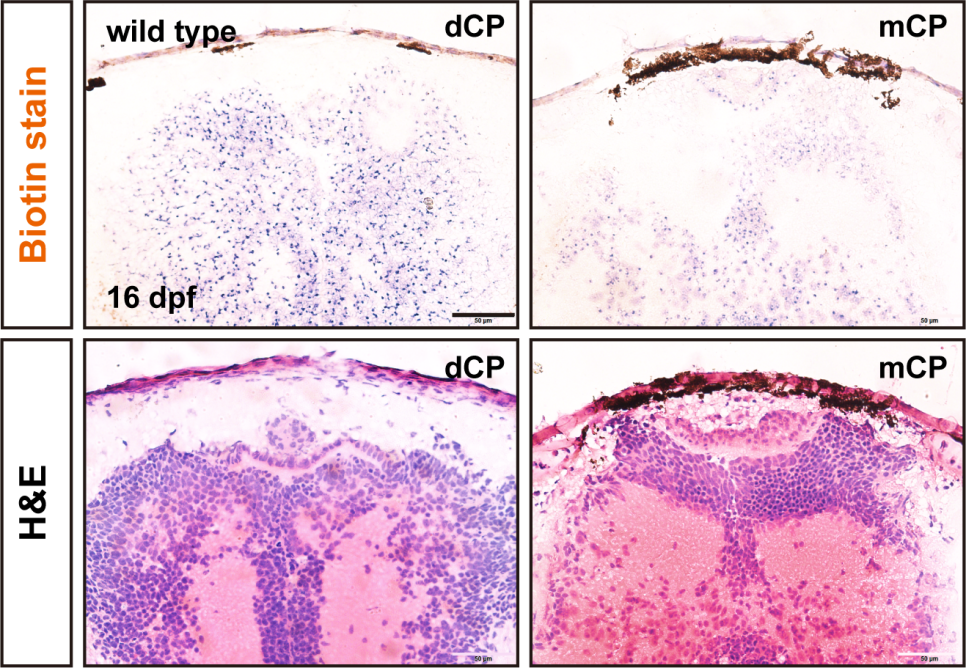


**Figure S8. Detection of endogenous biotin in zebrafish brain**. Biotin staining was performed to detect endogenous biotin in the brains of wild types at 16 dpf. Histological analysis by HE staining on adjacent slides of serial sections was performed to show the morphology of the brain. Scale bars, 50 μm.

**
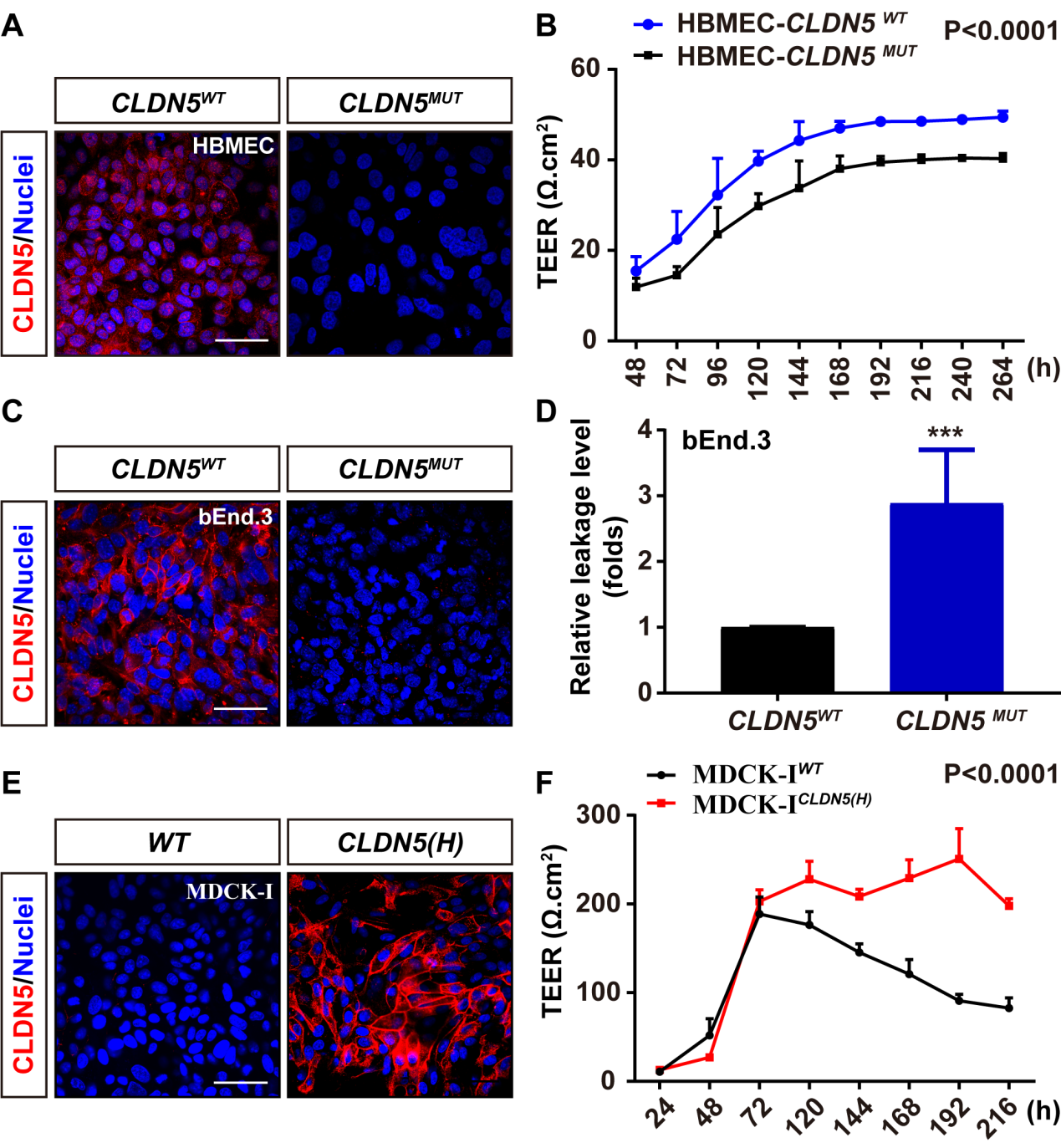
**

**Figure S9. CLDN5 is important for endothelial or epithelial barriers*.*** (A, B) Knock out of *CLDN5* in HBMEC cells (*CLDN5*^MUT^) causes a decrease in monolayer tightness as reflected by TEER measurement. (C, D) Knock out of *CLDN5* in bEnd.3 cells leads to an increased monolayer cell permeability as reflected by 10 KD-FITC dextran diffusion assay. (E, F) Expression of exogenous CLDN5 in MDCK-I cells (MDCK-I*^CLDN5(H)^*) efficiently reinforces the epithelial barrier function as reflected by TEER measurement. Scale bars, 50 μm. Each experiment was repeated for more than three times. Data are represented as mean ± SEM. *** P< 0.005.

**
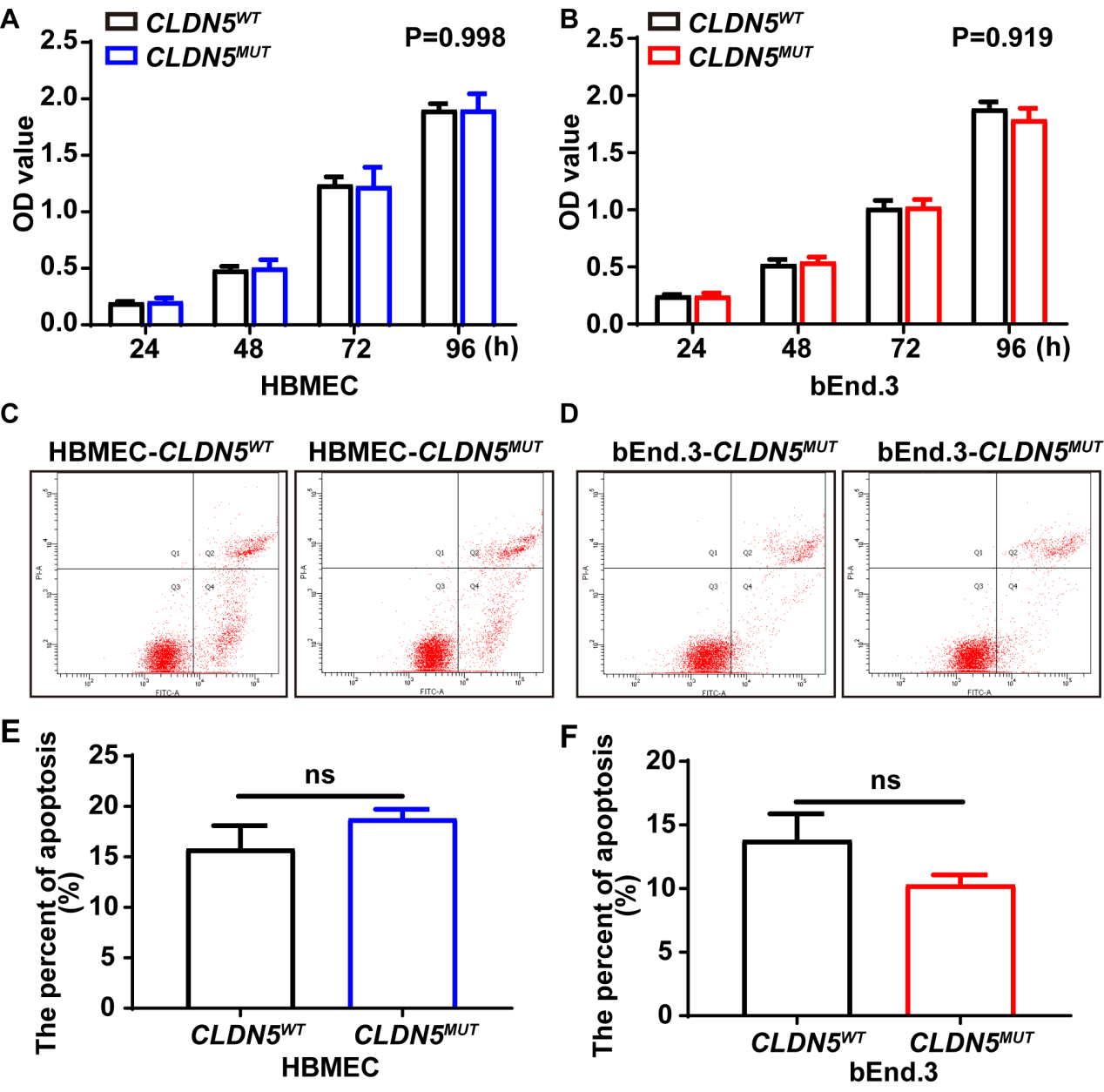
**

**Figure S10. Loss of CLDN5 in endothelial cells shows no effects on cell proliferation and apoptosis.** (A, B) CCK-8 assay was performed to detect the cell proliferation on HBMEC *CLDN5*^WT^ and HBMEC *CLDN5*^MUT^ cells, or bEnd.3 *CLDN5*^WT^ and bEnd.3 *CLDN5*^MUT^ cells at different time points of growth. (C-F) Cell apoptosis was measured by flow cytometry using Annexin V-FITC/PI staining. There is no statistic difference in the apoptosis between *CLDN5*^WT^ cells and *CLDN5*^MUT^ cells, for either HBMEC cells or bEnd.3 cells. Each experiment was repeated for more than three times. Data are represented as mean ± SEM. ns means no significance.

**
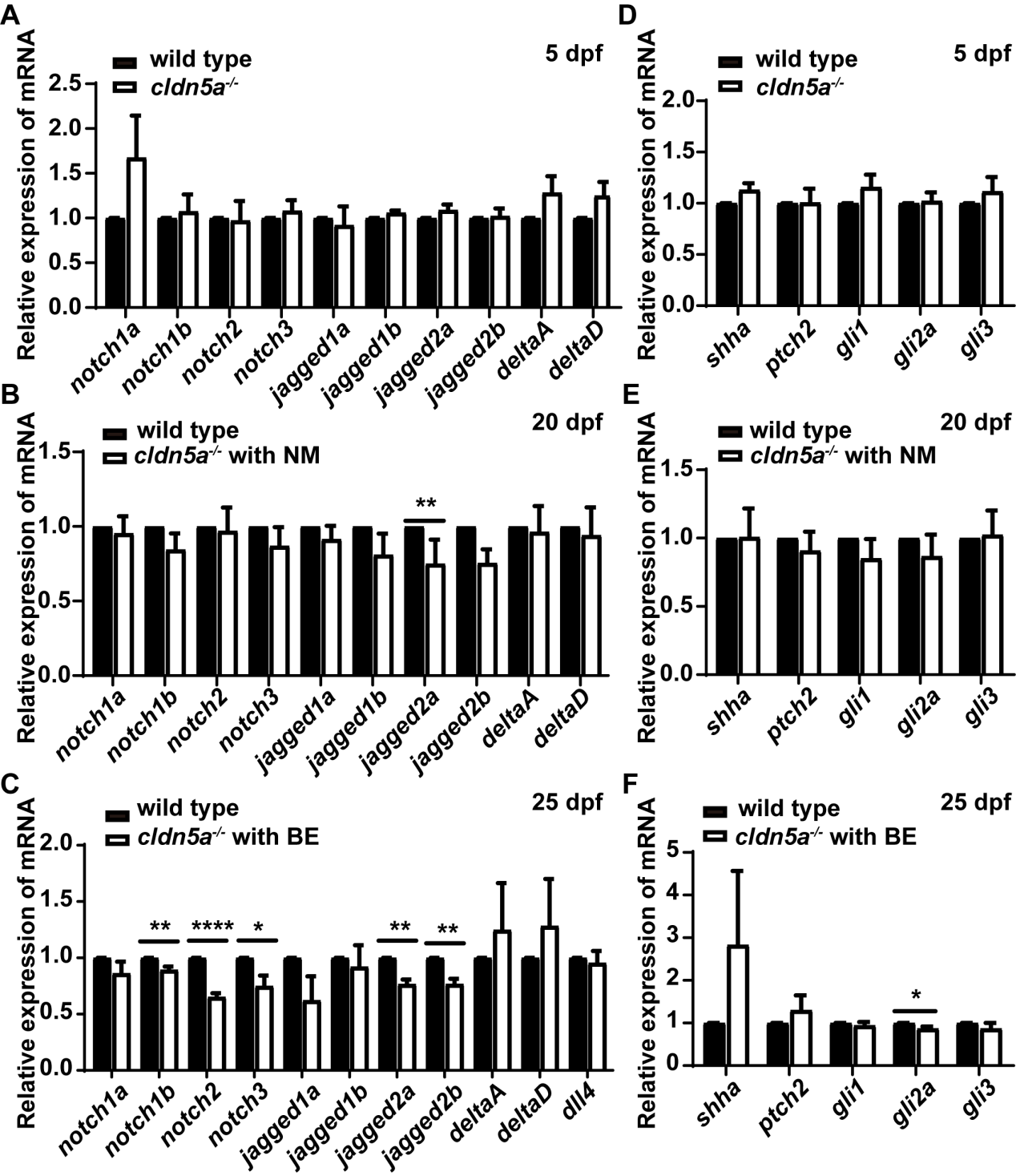
**

**Figure S11. The expression levels of genes for** **Notch and Shh pathways in *cldn5a^-/-^*** **are not affected.** (A-C) The expression levels of genes coding for typical factors of notch pathway were analyzed by RT-qPCR. (D-F) The expression levels of genes coding for key molecules of the shh pathway were analyzed by RT-qPCR. The brain samples were collected from embryos of 5 dpf (wild type and *cldn5a^-/-^*), larvae of 20 dpf- (wild type and *cldn5a^-/-^* with NM brain) or 25 dpf-old (wild type and *cldn5a^-/-^* with BE) respectively. Experiments were repeated for at least three times. Data are represented as mean ± SEM. *P<0.05, **P<0.01, ****P<0.001.

### Table S1. Primers used for PCR, RT-PCR, and RT-qPCR

| **Genes** | **Forward primer (5’-3’)** | **Reverse primer (5’-3’)** |
| --- | --- | --- |
| **PCR/RT-PCR** |  |  |
| *β-actin* (zebrafish) | CGTCTGGATCTAGCTGGTCGTGA | CAATTTCTCTTTCGGCTGTGGTG |
| *cldn5a* (zebrafish) | GTCTGATCCTGTGCGTCTG | CGCGTATTTGACCGAGTACC |
| *cldn5b* (zebrafish) | TTGGACTCTGCTTGACCGTC | CCATTGATCGATGTGCCTTT |
| **RT-qPCR** |  |  |
| *gapdh* (zebrafish) | AGAGGGACCCAGCCAACA | CAAAGGAGCCAGGCAGTT |
| *cldn5a* (zebrafish) | CGCTCACGGTCATCTCCTCG | ACAGTGGCACAAGCACGAAGA |
| *cldn5b* (zebrafish) | GTCATCTCTGCGGTTTTGGG | TTGGATTTGATAGACTCCGTGTTG |
| *il-6* (zebrafish) | TCAACTTCTCCAGCGTGATG | TCTTTCCCTCTTTTCCTCCTG |
| *il-8* (zebrafish) | GTCGCTGCATTGAAACAGAA | CTTAACCCATGGAGCAGAGG |
| *il-1β* (zebrafish) | TGGACTTCGCAGCACAAAATG | CACTTCACGCTCTTGGATGA |
| *il-4* (zebrafish) | AATGGGAAAGGGGAAAAAATGGAT | GTTTTCTTAGAGTAGTGTCC |
| *il-10* (zebrafish) | ATAAAACATAACATAAACAGTCCC | TGGCAGAATGGTCTCCAAGTA |
| *clusterin* (zebrafish) | GCCACCATCCAAAGAAGAGC | TTTCTCCATCTCCGACAGCA |
| *notch1a* (zebrafish) | CACGGAGCCAACCTGCACAA | GCACGACTCCAGCAGACGTT |
| *notch1b* (zebrafish) | GCAGAGCCAGAGCCAGAACT | CAGAGAGGACGGGTTGAGCA |
| *notch2* (zebrafish) | CATCACCACCAAGCCACCTA | AAGCCATCAGGAAGCCACAG |
| *notch3* (zebrafish) | CACTTACACCACCACAAGGAGA | TCATCATCTTCGGTCACCTCTG |
| *jagged1a* (zebrafish) | AGGCGGTCTGAGGCTAATCT | GTTCTCGTGGCAGTAGGTCC |
| *jagged1b* (zebrafish) | GCTCTGGATCTTCGCTCTGG | CTGCTCACGCACGTTATTGG |
| *jagged2a* (zebrafish) | TGCTCGCACACCTCACAA | CCTGTTGCTCTCCTCGTCTC |
| *jagged2b* (zebrafish) | ATGTAAACGGTGAGTTGTGGGA | TGGTGGTGACTTCAGACTGGTA |
| *deltaA* (zebrafish) | CCACTTGCCACGAAAGGAAC | ACACGCCACGACCAACAATA |
| *deltaD* (zebrafish) | CTGACAGTTGCGTCCTCCAT | GACGGACTCAGCGACTACAC |
| *dll4* (zebrafish) | CGGTTCATCTGCAACGAAAATTACT | TTCCAGCCAGGGAGACAGGATAA |
| *wnt2bb* (zebrafish) | GTTCCTTCTTCTCATCCTCACACCG | GTTCCTTCTTCTCATCCTCACACCG |
| *wnt3a* (zebrafish) | CATAATGTGTAGCTCAATTCCTGGC | TGTCGTTGATAGTAGTGCAGTTCCA |
| *wnt8b* (zebrafish) | ACAACGAAGTAGGACGCAAGG | CTTTTAGGTAGTTTCCCACTTCTCG |
| *gsk3b* (zebrafish) | GCAGAGATAAAGATGGCAGTAAAGT | AAAGAGCCGTTACCGATGACC |
| *beta-catenin* (zebrafish) | ATGAGGGCATGCAGATACCTTCCA | TTGACCACGGCATGTTTGAGCATC |
| *axin2* (zebrafish) | AATCTTTGCATTTTCTTCTCGGGG | CTCTGTGCGTTTTGGTATCCTT |
| *shha* (zebrafish) | TCGCCTACAAGCAGTTCATACC | TCCCGTGTTCTCCTCATCCTTA |
| *ptch2* (zebrafish) | AGTCCTTCTCCTGAGCCCAT | CAGAGGTGGTCGTCGTTTCA |
| *gli1* (zebrafish) | GATACACATGCACCACGACCTA | TCAATCTGAGCGTTCTCCAAGT |
| *gli2a* (zebrafish) | TGGCATCTCCTGGTGTCAAC | CGAATGGTCTCCGTCATCCA |
| *gli3* (zebrafish) | ACTTCCTCAGTCCACAGCAG | AACATCGGCACTACCAGAGC |

### Table S2. Primary and secondary antibodies used for IHC and IF staining

| **Primary and secondary antibodies** | **Diluted rate** | **CAT.No. and Company** |
| --- | --- | --- |
| Mouse anti-Cldn5 antibody | 1:150 | 35-2500, ThermoFisher Scientific |
| Rabbit anti-Cldn5 antibody | 1:150 | 34-1600, ThermoFisher Scientific |
| Rabbit anti-Cldn1 antibody | 1:150 | 51-9000, ThermoFisher Scientific |
| Rabbit anti-Cldn3 antibody | 1:150 | 187340, ThermoFisher Scientific |
| Rabbit anti-PKC ζ (C-20) antibody | 1:150 | sc-216, Santa Cruz Biotechnology |
| Rabbit anti-ZO-1 antibody | 1:150 | 40-2200, ThermoFisher Scientific |
| Mouse anti-Beta-catenin antibody | 1:150 | 13-8400, ThermoFisher Scientific |
| Alexa Fluor® 647 AffiniPure Goat Anti-Rabbit IgG (H+L) | 1:150 | 111-605-003, Jackson ImmunoResearch |
| Fluorescein (FITC) AffiniPure Goat Anti-Mouse IgG (H+L) | 1:150 | 115-095-003, Jackson ImmunoResearch |
